# Impact of personal genomic risk information on melanoma prevention behaviors and psychological outcomes: a randomized controlled trial

**DOI:** 10.1038/s41436-021-01292-w

**Published:** 2021-08-12

**Authors:** Amelia K. Smit, Martin Allen, Brooke Beswick, Phyllis Butow, Hugh Dawkins, Suzanne J. Dobbinson, Kate L. Dunlop, David Espinoza, Georgina Fenton, Peter A. Kanetsky, Louise Keogh, Michael G. Kimlin, Judy Kirk, Matthew H. Law, Serigne Lo, Cynthia Low, Graham J. Mann, Gillian Reyes-Marcelino, Rachael L. Morton, Ainsley J. Newson, Jacqueline Savard, Lyndal Trevena, Sarah Wordsworth, Anne E. Cust

**Affiliations:** 1 The Daffodil Centre, The University of Sydney, a joint venture with Cancer Council NSW, NSW Sydney, Australia; 2 Melanoma Institute Australia, The University of Sydney, Sydney, NSW Australia; 3 Electrical and Computer Engineering, University of Canterbury, Christchurch, New Zealand; 4 Centre for Medical Psychology and Evidence-based Decision-making, School of Psychology, The University of Sydney, Sydney, NSW Australia; 5 Division of Genetics, School of Biomedical Sciences, University of Western Australia, Crawley, WA Australia; 6 School of Medicine, The University of Notre Dame, Notre Dame, NSW Australia; 7 Cancer Council Victoria, Melbourne, VIC Australia; 8 NHMRC Clinical Trials Centre, The University of Sydney, Sydney, NSW Australia; 9 H. Lee Moffitt Cancer Center and Research Institute, Tampa, FL USA; 10 Melbourne School of Population and Global Health, The University of Melbourne, Parkville, VIC Australia; 11 Queensland University of Technology, School of Biomedical Sciences, Brisbane, QLD Australia; 12 Westmead Clinical School and Westmead Institute for Medical Research, Sydney Medical School, The University of Sydney, Sydney, NSW Australia; 13 Statistical Genetics, QIMR Berghofer Medical Research Institute, Brisbane, QLD Australia; 14 Queensland University of Technology (QUT), Brisbane, QLD Australia; 15 Consumer representative, Brisbane, QLD Australia; 16 The John Curtin School of Medical Research, ANU College of Health and Medicine, ANU, ACT, Canberra, Australia; 17 Sydney Health Ethics, Sydney School of Public Health, The University of Sydney, Sydney, NSW Australia; 18 School of Medicine, Faculty of Health, Deakin University, Geelong, VIC Australia; 19 Sydney School of Public Health, The University of Sydney, Sydney, NSW Australia; 20 Health Economics Research Centre, The University of Oxford, Oxford, UK

## Abstract

**Purpose:**

We evaluated the impact of personal melanoma genomic risk information on sun-related behaviors and psychological outcomes.

**Methods:**

In this parallel group, open, randomized controlled trial, 1,025 Australians of European ancestry without melanoma and aged 18–69 years were recruited via the Medicare database (3% consent). Participants were randomized to the intervention (*n* = 513; saliva sample for genetic testing, personalized melanoma risk booklet based on a 40-variant polygenic risk score, telephone-based genetic counseling, educational booklet) or control (*n* = 512; educational booklet). Wrist-worn ultraviolet (UV) radiation dosimeters (10-day wear) and questionnaires were administered at baseline, 1 month postintervention, and 12 months postbaseline.

**Results:**

At 12 months, 948 (92%) participants completed dosimetry and 973 (95%) the questionnaire. For the primary outcome, there was no effect of the genomic risk intervention on objectively measured UV exposure at 12 months, irrespective of traditional risk factors. For secondary outcomes at 12 months, the intervention reduced sunburns (risk ratio: 0.72, 95% confidence interval: 0.54–0.96), and increased skin examinations among women. Melanoma-related worry was reduced. There was no overall impact on general psychological distress.

**Conclusion:**

Personalized genomic risk information did not influence sun exposure patterns but did improve some skin cancer prevention and early detection behaviors, suggesting it may be useful for precision prevention. There was no evidence of psychological harm.

## INTRODUCTION

Primary and secondary prevention are crucial for skin cancer control. Most melanomas and other skin cancers are caused by ultraviolet radiation (UV) and are largely preventable through sun protection behaviors [1, 2], and early detection is associated with improved prognosis [3, 4].

The US Preventive Services Task Force has called for research targeting high-risk groups for skin cancer screening, surveillance, and behavioral counseling [5, 6]. Genomic risk is one approach to stratifying people according to personal risk. Common genomic variants each have small to moderate effect sizes for melanoma risk, and when aggregated in a polygenic risk score they have been shown to be as good as, or better than, other more traditional measures of melanoma risk such as skin type or family history [7]. Genomic risk may also be easier to measure and can identify individuals at high risk despite an absence of traditional, often visible, risk factors [7].

Providing personal melanoma genomic risk information based on a polygenic risk score might motivate prevention behaviors in the general population, but there is limited evidence to support it. Most studies of genomic risk interventions in healthy participants have focused on smoking cessation, diet, and physical activity behaviors, and have been limited by small sample sizes, a high risk of bias, selected settings, and single or few genomic variants [8]. The Multiplex Initiative [9] included skin cancer as one of eight health conditions for the evaluation of genetic susceptibility testing but did not evaluate skin cancer prevention outcomes. Trials in Florida and New Mexico have assessed feedback of melanoma risk information in primary care settings based on carriage of *MC1R* genotype alone, although the behavioral outcomes are not yet published [10, 11]. Some studies have focused on providing information on rarer high-risk, single-gene variants to melanoma-prone families and shown increased motivation to improve sun-related behaviors [12, 13]. Providing genetic risk information about melanoma in these specialized contexts may well be expected to provide different outcomes than providing personalized genomic risk information to the general population. Nonetheless, tailored melanoma risk information based on *traditional* risk factors, not genotype information, targeted at people at moderate or high melanoma risk have also shown improvements in skin cancer-related behaviors [14, 15].

We conducted the Melanoma Genomics Managing Your Risk Study, a large, population-based randomized controlled trial to evaluate our hypothesis that giving the public information on personal genomic risk of melanoma based on a polygenic risk score would motivate reduced sun exposure, and increased sun protection and early detection behaviors, with no psychological harms. We also hypothesized that the behavioral effect may differ according to the presence of traditional risk factors, such as sun-sensitive phenotype or family history of melanoma, as these factors may influence sun-related preventive behaviors and risk perception [16, 17].

## MATERIALS AND METHODS

### Study design

**Fig. 1:**
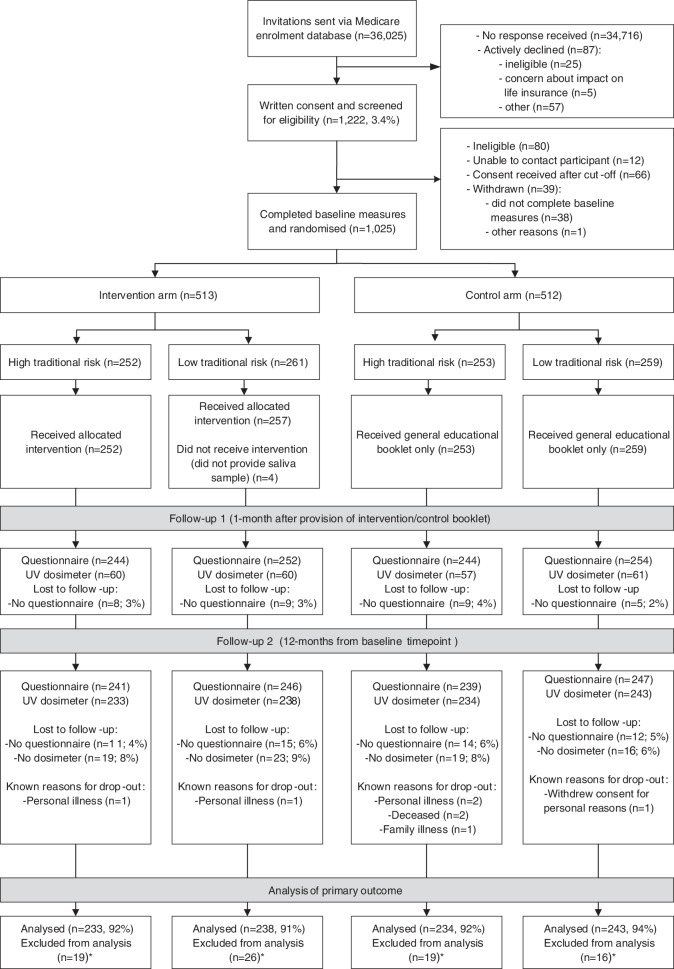
CONSORT flow diagram. *Participants excluded from analysis of primary outcome where they had no ultraviolet (UV) dosimeter data.

The study protocol and statistical analysis plan have been previously published [18, 19]. We provide brief details here, according to CONSORT guidelines. We used a randomized controlled trial design (Fig. 1). The study was conducted in Australia, a country with high ambient UV and the highest melanoma incidence in the world [20]. The theoretical foundation of the study design and measures draws on constructs from protection motivation theory and the health belief model, which help explain how health information may influence protective behaviors; this is described in more detail in the published protocol [19]. Ethical approval was obtained from the Human Research Ethics Committee at The University of Sydney (2017/163) and all participants gave informed consent.

### Participants

Eligible participants met the following criteria:

1. Aged 18–69 years at the time of recruitment. This age group was selected to maximize the impact of the intervention, in relation to primary prevention behaviors preventing future melanoma and other skin cancers, and skin checks leading to early detection and better prognosis. Participants ≥70 years were not included because there is less capacity to impact future melanoma incidence and they are more likely to have comorbidities that may influence time outdoors.
2. No personal history of melanoma, since this will alter personal risk estimates and behaviors.
3. Full or part European ancestry, as genomic risk estimates for melanoma have been derived from populations with European ancestry and so may be less accurate for other ethnicities.

Recruitment took place during the spring/summer seasons between October 2017 and February 2019, with 12-month follow-up also during spring/summer from October 2018 to March 2020. Potential participants aged 18–69 years, sampled to be representative of Australians’ State and Territory locations of residence, were sent a study invitation pack via the Australian Government’s Department of Human Services’ Medicare database (see Supplementary Methods for more recruitment details).

### Traditional risk scores

At baseline, traditional risk scores were calculated based on a validated melanoma risk prediction model that included moles (nevi), hair color, artificial sunbed use, first-degree blood relative(s) with melanoma, and personal history of keratinocyte cancers [21]; the scores were dichotomized as high or low based on the approximate midpoint score [18]. Participants were not provided with their traditional risk category as we were specifically interested in evaluating the impact of genomic risk information. Previous studies have shown that sun-sensitive phenotype, family history, and skin cancer risk perception are associated with sun protection and skin check behaviors (although there is variability in findings) [16, 17] and that self-perceived risk of developing melanoma is weakly related to actual risk [22].

### Randomization and masking

After participants completed the baseline measures (questionnaire and UV dosimeter), they were randomized to the intervention or control arm (allocation ratio 1:1) by the University of Sydney’s National Health and Medical Research Council (NHMRC) Clinical Trials Center Randomization Service using a computer-based system. A minimization procedure was used to balance the study groups by traditional risk (low, high), gender, state of residence, and age group (18–44, 45–69 years). Staff managing the study were not involved in the randomization process, and vice versa. Participants and staff were not blinded to study group allocation.

### Procedures

#### Intervention arm

Participants randomized to the intervention arm received:

1. A mailed “at home” saliva collection kit (Oragene® DNA Genotek) for DNA collection and genotyping.
2. A personalized booklet describing their personal genomic risk of melanoma, calculated from a validated polygenic risk score based on 40 variants in 20 genes/gene regions with established associations with melanoma risk [23], and underlying age/sex/state-specific population melanoma incidence and competing mortality rates (more details in the protocol [19]). Participants had the option to also have a summary sent to their general practitioner (GP).
3. A telephone call from the study genetic counselor within two weeks of posting their personalized booklet to confirm booklet receipt, discuss their genomic risk results, and answer any participant questions. Traditional risk factors such as previous sunburns and family history would also influence an individual’s risk, and this was emphasized to participants in the booklets and during the phone call to avoid giving false reassurance based only on personal genomic risk.
4. A general educational booklet with information on melanoma preventive behaviors such as staying safe in the sun, using sun protection, and conducting skin examinations.

#### Control arm

Participants randomized to the control arm received only the general educational booklet. Educational materials are widely available in Australia and so were considered standard care.

### Outcomes

Brief details of the outcomes and measures used are described below; full details are provided in the published statistical analysis plan [18] and study protocol [19].

#### Timing and administration of outcome measures

Baseline measures were captured before randomization. Follow-up 1 measures occurred 1 month after the provision of intervention/control booklets; this was on average 3 months after baseline, and the timing was matched for control and intervention participants (and by state and gender) to ensure no seasonal or weather differences between groups. Follow-up 2 was captured 12 months after baseline assessments. Questionnaire measures were administered online using a REDCap database hosted by the University of Sydney at baseline, follow-up 1 and follow-up 2. All participants were asked to wear an electronic UV dosimeter during daylight hours for 10 days at baseline and follow-up 2. They were mounted in lightweight custom-made wristbands attached to the left wrist. A subgroup (*n* = 238) was also asked to wear a UV dosimeter at follow-up 1. The UV dosimeters were used to measure time-stamped UV exposure and did not provide any feedback to participants.

#### Primary outcome measure

The primary outcome was total daily personal standard erythemal doses (SEDs) at 12 months after baseline, measured using the UV dosimeters.

#### Secondary outcome measures

Secondary outcomes were sourced from validated instruments and other studies where possible, and included:

1. Objectively measured time-specific SEDs during (1) peak UV hours (between 10 am and 2 pm or 11 am and 3 pm local time, depending on Daylight Savings Time), (2) morning (6 am–10 am or 7 am–11 am) and (3) afternoon (2 pm–6 pm or 3 pm–7 pm) periods.
2. Self-reported sun exposure measured as time spent outdoors on a typical weekday and a typical weekend during the past month, measured in 15-minute intervals (0, <15, 15–29, 30–44, 45–60 minutes) between 7 am and 6 pm. Total exposure time was calculated using the sum of the midpoint times.
3. Six sun protection behaviors [24] (sunscreen use, wearing a shirt with sleeves, wearing a hat, seeking shade, wearing sunglasses, limiting peak time sun exposure) were measured using a Likert scale (1 = never/rarely, 2 = sometimes, 3 = often, 4 = always) over the past month. Behaviors were analyzed as a composite mean score, and individually.
4. Whole-body skin examinations, combining skin examinations performed oneself or by a partner or health professional.
5. Outdoor intentional tanning frequency (1 = never, 2 = rarely, 3 = sometimes, 4 = often, 5 = always) over the past month.
6. Sunburn frequency recalled over the previous month (0, 1, 2, 3 or more times).
7. Melanoma-related worry, using three items measured on a Likert scale (1 = not at all, 2 = rarely, 3 = sometimes, 4 = often, 5 = almost all the time) [25, 26].
8. General psychological distress using the 5-item version of the Mental Health Inventory (MHI-5) designed for primary care settings [27].
9. Intervention arm only: the impact of the receipt of personal genomic risk (categorized as low, average, or high) on the outcomes above, and on genetic testing–specific distress, uncertainty and positive experiences measured at follow-up 1 and follow-up 2 using the Multidimensional Impact of Cancer Risk Assessment (MICRA) [28].

### Statistical analysis

The target sample size of 892 people (446 per arm) was calculated using: ratio = 0.8, variation = 0.9, alpha = 0.05 [18]. This was based on detecting a 20% difference in the geometric mean of daily SEDs between intervention and control arms, separately for low and high traditional risk groups, and allowing up to 15% loss to follow-up [19].

All intervention (genomic risk) versus control (usual care) group comparisons conducted for the primary and secondary outcomes were intention-to-treat, two-tailed with a nominal 5% significance level, and adjusted for baseline scores and randomized stratification factors (gender, state of residence, age group). These analyses were presented stratified by traditional risk (high, low) as it was hypothesized that the effect of the genomic risk intervention may differ by traditional risk factors (due to their relation to a person’s underlying risk perception and behaviors). For analyses that pooled traditional risk groups, the model also fitted a variable for traditional risk group. More details, including for subgroup analyses, are provided in the published statistical analysis plan [18] and in the Supplementary text.

## RESULTS

### Participation and participant characteristics

Figure 1 shows participant flow through the trial process. A total of 1,025 participants were randomized (intervention, *n* = 513; control, *n* = 512). Of the total invited sample, 3.4% gave consent and 2.8% were randomized, but differed according to age and gender: 1.3% randomized for men and 3.0% for women aged 18–44 years, and 4.5% for men and 6.7% for women aged 45–69 years. Table 1 shows the values for the randomization stratification variables at baseline, by trial arm and traditional risk group. Other baseline characteristics are shown in Supplementary Table 1.

**Table 1:** Randomization factors by study arm, stratified by traditional risk groups.

	High traditional risk (*n* = 505)			Low traditional risk (*n* = 519)	
	Intervention	Control		Intervention	Control
	(*n* = 252)	(*n* = 253)		(*n *= 261)	(*n* = 258)^a^
Females *N*, (%)	131 (52.0%)	135 (53.4%)		130 (49.8%)	126 (48.8%)
Age group *N*, (%)					
18–44 years	108 (42.9%)	117 (46.3%)		133 (51.0%)	125 (48.5%)
45–69 years	144 (57.1%)	136 (53.8%)		128 (49.0%)	133 (51.6%)
Age in years, mean, (SD)	48.2 (14.0)	47.9 (13.9)		46.0 (15.4)	45.5 (14.6)
State *N*, (%)					
NSW	87 (34.5%)	79 (31.2%)		59 (22.6%)	64 (24.8%)
QLD	53 (21.0%)	65 (25.7%)		52 (19.9%)	40 (15.5%)
WA	23 (9.1%)	22 (8.7%)		31 (11.9%)	30 (11.6%)
NT	2 (0.8%)	3 (1.2%)		0 (0%)	1 (0.4%)
TAS	8 (3.2%)	12 (4.7%)		14 (5.4%)	11 (4.3%)
VIC	66 (26.2%)	62 (24.5%)		79 (30.3%)	86 (33.3%)
SA	8 (3.2%)	6 (2.4%)		22 (8.4%)	20 (7.8%)
ACT	5 (2.0%)	4 (1.6%)		4 (1.5%)	6 (2.3%)

^a^Excluding one participant who withdrew all consent from the study.

The distribution of personal genomic risk estimates for the intervention arm are summarized in Supplementary Figure 1A and 1B. Of participants in the intervention arm, 95% consented to having a summary of their personal genomic risk information sent to their GP. Intervention process measures, including results showing the uptake, recall, satisfaction, and understanding of the intervention, are shown in Supplementary Tables 2 and 3, and Supplementary Results.

At follow-up 1, 994 (97%) participants completed the questionnaire and dosimetry was completed by a subset of 238 (23%) participants. At follow-up 2, 973 (95%) participants completed the questionnaire and 948 (92%) completed dosimetry. Descriptive statistics for primary and secondary outcomes are shown in Supplementary Table 4. Participants were asked to wear the dosimeter on weekdays and weekends (achieved for 99% of participants). Participants wore the dosimeters for an average of seven weekdays (standard deviation [SD]: two days) and three weekend days (SD: one day) at each time point, which was similar by gender and age group.

### Primary outcome: objective measure of UV exposure

**Fig. 2:**
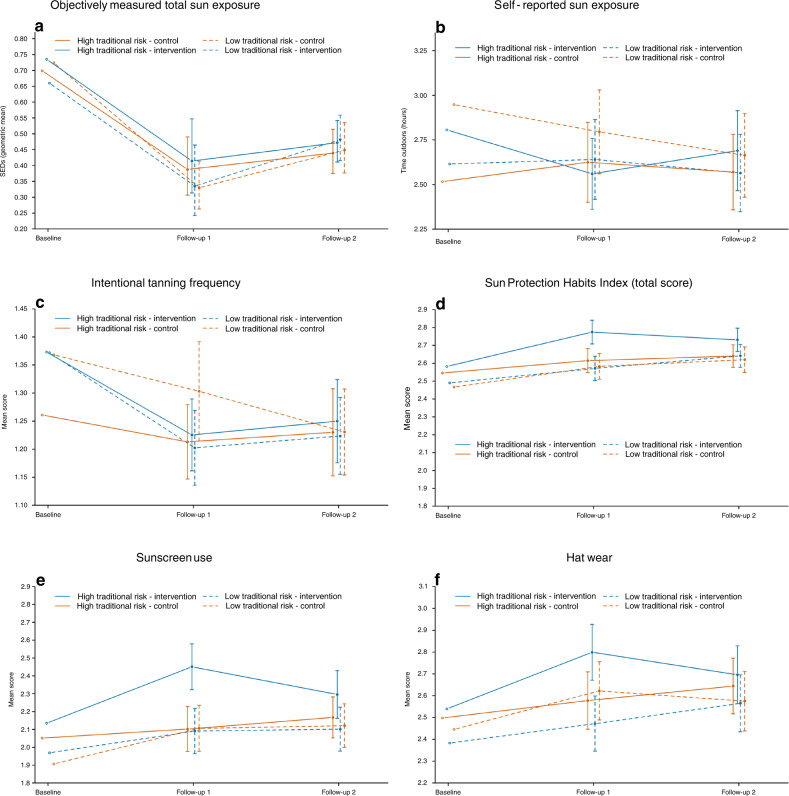
(a) Objectively measured daily standard erythemal doses (SEDs) at baseline, follow-up 1 and 2 for intervention and control arms, stratified by traditional risk groups. Vertical bars indicate 95% confidence intervals (CI). At follow-up 1, the percentage difference and 95% CI comparing intervention with control arms in the low traditional risk group was 4.60% (95% CI: −5.08, 15.27; *p* = 0.36) and 2.49% (95% CI: −7.94, 14.14; *p* = 0.65) in the high traditional risk group. At follow-up 2, the percentage differences were 1.03% (95% CI: −4.84, 7.26; *p* = 0.74) and −1.44 (95% CI: −6.89 to 4.33, *p *= 0.62), respectively. (**b**) Self-reported daily total time spent outdoors at baseline, follow-up 1 and 2 for intervention and control arms, stratified by traditional risk groups. Vertical bars indicate 95% CI. At follow-up 1, the mean difference comparing intervention with control arms in the low traditional risk group was 0.07 (95% CI: −0.15, 0.30; *p* = 0.53) and −0.21 (95% CI: −0.43, 0.02; *p* = 0.07) in the high traditional risk group. At follow-up 2, the mean differences were 0.17 (95% CI: −0.06, 0.39; *p* = 0.15) and −0.03 (95% CI: −0.26, 0.20; *p* = 0.79), respectively. (**c**) Intentional tanning frequency (mean score of 1 item on Likert scale 1_never_—5_always_) at baseline, follow-up 1 and 2 for intervention and control arms, stratified by traditional risk groups. Vertical bars indicate 95% CI. At follow-up 1, the mean difference comparing intervention with control arms in the low traditional risk group was −0.10 (95% CI: −0.19, −0.01; *p* = 0.03) and −0.03 (95% CI: −0.11, 0.06; *p* = 0.53) in the high traditional risk group. At follow-up 2, the mean differences were −0.00 (95% CI: −0.09, 0.09; *p* = 1.00) and −0.02 (95% CI: −0.11, 0.06; *p* = 0.62), respectively. (**d**) Sun Protection Habits Index (mean score of Likert scale: 1_never/rarely_—4_always_, for six sun protection behaviors) at baseline, follow-up 1 and 2 for intervention and control arms, stratified by traditional risk groups. Vertical bars indicate 95% CI. At follow-up 1, the mean difference comparing intervention with control arms in the low traditional risk group was −0.03 (95% CI: −0.10, 0.04; *p* = 0.43) and 0.12 (95% CI: 0.05, 0.19; *p *= <0.001) in the high traditional risk group. At follow-up 2, the mean differences were −0.01 (95% CI: −0.08, 0.06; *p* = 0.83) and 0.06 (95% CI: −0.01, 0.13; *p *= 0.08), respectively. (**e**) Sunscreen use (one item from the Sun Protection Habits Index; mean score of Likert scale: 1_never/rarely_—4_always_) at baseline, follow-up 1 and 2 for intervention and control arms, stratified by traditional risk groups. Vertical bars indicate 95% CI. At follow-up 1, the mean difference comparing intervention with control arms in the low traditional risk group was −0.06 (95% CI: −0.19, 0.08; *p* = 0.41) and 0.28 (95% CI: 0.15, 0.42; *p* = <0.001) in the high traditional risk group. At follow-up 2, the mean differences were -0.07 (95% CI: −0.21, 0.06; *p* = 0.28) and 0.08 (95% CI: −0.06, 0.22; *p* = 0.26), respectively. (**f**) Hat use (one item from the Sun Protection Habits Index; mean score of Likert scale: 1_never/rarely_—4_always_) at baseline, follow-up 1 and 2 for intervention and control arms, stratified by traditional risk groups. Vertical bars indicate 95% CI. At follow-up 1, the mean difference comparing intervention with control arms in the low traditional risk group was −0.11 (95% CI: −0.24, 0.01; *p* = 0.08) and 0.16 (95% CI: 0.04, 0.29; *p* = 0.01) in the high traditional risk group. At follow-up 2, the mean differences were 0.01 (95% CI: −0.12, 0.14; *p* = 0.90) and 0.00 (95% CI: −0.12, 0.13; *p* = 0.97), respectively.

Comparing participants in the intervention versus control arms, there was no difference in the primary outcome of total daily dosimeter-measured UV exposure (SEDs) at follow-up 2, in either the low (percentage difference: 1.03%, 95% CI: −4.84, 7.26; *p* = 0.74) or high (−1.44, 95% CI: −6.89 to 4.33; *p* = 0.62) traditional risk groups (Fig. 2a). Participants in both trial arms decreased their time in the sun during the study (*p* <0.001; Supplementary Table 4).

In subgroup analyses of the primary outcome, there was an interaction according to genetic determinism, i.e., the extent to which genetic makeup determines whether or not a person will develop melanoma (*p*-interaction 0.0008). The intervention reduced SEDs by a mean 7.56% (95% CI: −2.05, −12.75 *p* = 0.008) for the intervention versus control arm among those with weak deterministic views, but increased SEDs by a mean 6.46% (95% CI: 0.48, 12.81, *p *= 0.03) among those with strong deterministic views (Supplementary Figure 2). The interaction effect by genetic determinism was similar for low and high traditional risk groups.

### Secondary outcomes

#### Self-reported time spent outdoors and objectively measured time-specific UV exposure

There was no effect of the intervention on dosimeter-measured UV exposure at different times of day, nor on self-reported total daily sun exposure (Fig. 2b) or peak UV time sun exposure.

#### Intentional tanning

The intervention reduced intentional tanning frequency at follow-up 1 overall (mean score difference between intervention and control: −0.06, 95% CI: −0.13, 0.00; *p* = 0.04) and among the low traditional risk group (Fig. 2c), but this was attenuated at follow-up 2. There was an interaction of the intervention effect by gender (*p*-interaction 0.02); among women the intervention reduced intentional tanning frequency (at follow-up 1 −0.12, 95% CI: −0.22, −0.03; *p* = 0.01) but not among men (0.00; 95% CI: −0.08, 0.08; *p *= 1.0). For both the intervention and control arms, women had higher intentional tanning frequency at baseline compared to men.

#### Sun protection

The intervention improved self-reported sun protection behaviors at follow-up 1 among the high traditional risk group (mean difference in the total index 0.12, *p* <0.001), particularly sunscreen use (0.28, *p* <0.001) and wearing a hat (0.16, *p* = 0.01), but this was attenuated at follow-up 2 (Figs. 2d, e, f). The effects of the intervention were stronger for the high traditional risk group than for the low traditional risk group (*p*-interaction = 0.045 for the total index, 0.02 for sunscreen, 0.07 for hat wear).

Comparing the intervention with control arms within different population subgroups, the intervention improved sun protection behaviors among older participants (*p*-interaction = 0.02) and those residing in an area with higher socioeconomic indicators (*p*-interaction = 0.03). At 12 months follow-up, the adjusted mean difference in sun protection index between intervention and control groups aged 45–69 years was 0.07 (95% CI: 0.00, 0.13; *p* = 0.06) overall and 0.14 (95% CI: 0.05, 0.24; *p* = 0.004) among the older high traditional risk group.

#### Sunburn

**Fig. 3:**
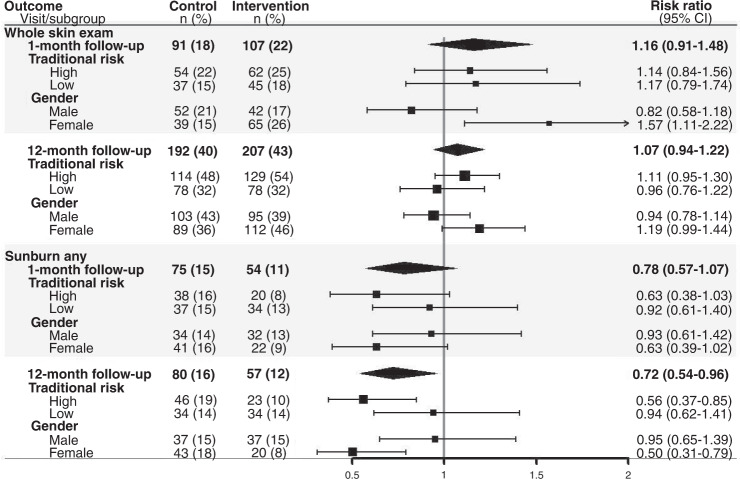
Relative risks and 95% confidence intervals for whole-body skin examinations (any versus none; clinical or self-conducted) and sunburns (any versus none) at 1- and 12-month follow-up comparing intervention with control, stratified by traditional risk groups and gender. The black vertical line is the line of no effect (i.e., the position at which there is no clear difference between study groups). Estimates to the right of the black vertical line indicate that the event (skin examinations or sunburns) occurred more frequently in the intervention group than the control group, and estimates to the left of the black vertical line indicate that the event occurred less frequently in the intervention group than the control group.

The intervention reduced self-reported sunburn incidence in the intervention versus control group by 28% (relative risk 0.72, 95% CI: 0.54, 0.96; *p *= 0.02) at 12 months, and the effect was particularly evident among the high traditional risk group (*p*-interaction = 0.06) and among women (*p*-interaction = 0.05) (Fig. 3).

#### Skin examinations

For whole-body skin examinations, there was no effect of the intervention overall or stratified by traditional risk at follow-up 1 or 2. However, when stratified by gender, the intervention increased skin examinations among women but not men overall (*p*-interaction 0.045) (Fig. 3), and within the high traditional risk group where the relative risk for skin examinations at 12 months was 1.33 (95% CI: 1.06, 1.67) among women and 0.95 (95% CI: 0.76, 1.18) among men (*p*-interaction 0.04).

#### Psychological outcomes

The intervention reduced melanoma-related worry at 12 months, overall (mean difference -0.09, 95% CI: -0.15, -0.03; *p* = 0.002) and within low and high traditional risk groups (Supplementary Figure 3A). This reduction was observed for people with higher health literacy but not lower health literacy (*p*-interaction = 0.03). There was no impact of the intervention on general psychological distress (MHI-5) overall or according to traditional risk groups (Supplementary Figure 3B). When stratified by gender, there was an interaction such that the intervention lowered general distress among men but not women particularly at follow-up 1 (*p*-interaction = 0.03).

### Outcomes by genomic risk category among the intervention arm only

Participants in the intervention arm who received a higher than average genomic risk result reported a greater increase in sun protection behaviors than those in the low genomic risk category at follow-up 1 (*p* = 0.005 for the total score) but not 12 months follow-up (Table 2). Participants who received a low genomic risk result did not worsen their sun protection behaviors (mean sun protection index score was 2.50 at baseline, 2.58 at follow-up 1, and 2.69 at follow-up 2) or sun exposure (geometric mean SEDs 0.60 at baseline and 0.46 at follow-up 2). Melanoma-related worry scores reduced in the low genomic risk group (1.93, 1.84, 1.74 at baseline, follow-up 1 and 2) and remained constant in the high genomic risk group (1.97, 1.97, 1.93, respectively), leading to a statistically significant difference between groups. The effect of the intervention on UV dosimetry and skin examinations did not differ by genomic risk group. Assessment using the MICRA instrument showed low levels of psychological distress and uncertainty and high scores for positive experiences for all genomic risk groups, but there was a consistent gradient in the scores showing increasing mean values corresponding with higher genomic risk (Supplementary Table 5).

**Table 2:** Outcomes in the intervention arm only, stratified by genomic risk category.

	Follow-up 1 or 2	Intervention effect^a^ (95% confidence interval) for the average and high genomic risk groups relative to the low genomic risk group (reference category)			
		Low genomic risk	Average genomic risk	High genomic risk	*P* value^c^
		*n* = 104/104^b^	*n* = 264/256^2^	*n* = 127/126^2^	
Behavioral outcome—objectively measured			% difference (95% CI)	% difference (95% CI)	
UV exposure, SEDs/day	1	Ref	−8.80 (22.10, 6.78)	−6.99 (−22.06, 10.99)	0.38
	2	Ref	3.79 (−4.51, 12.81)	−0.58 (−9.55, 9.26)	0.34
Behavioral outcomes—self-reported			Mean difference (95% CI)	Mean difference (95% CI)	
Sun exposure, total hours/day	1	Ref	0.00 (−0.28, 0.28)	−0.06 (−0.38, 0.26)	0.89
	2	Ref	0.02 (−0.26, 0.30)	−0.15 (−0.47, 0.17)	0.41
Sun Protection Index (total score); 1_never/rarely_–4_always_	1	Ref	0.05 (−0.03, 0.14)	0.16 (0.06, 0.25)	0.005
	2	Ref	−0.07 (−0.16, 0.02)	0.02 (−0.08, 0.12)	0.07
Limit midday sun exposure	1	Ref	−0.07 (−0.24, 0.10)	0.15 (−0.04, 0.35)	0.02
	2	Ref	−0.07 (−0.24, 0.11)	0.01 (−0.19, 0.21)	0.58
Stay in shade	1	Ref	0.09 (−0.07, 0.26)	0.13 (−0.07, 0.32)	0.41
	2	Ref	0.00 (−0.16, 0.17)	0.09 (−0.10, 0.28)	0.54
Wear a hat	1	Ref	0.08 (−0.08, 0.24)	0.13 (−0.05, 0.32)	0.36
	2	Ref	−0.04 (−0.20, 0.13)	0.10 (−0.09, 0.28)	0.22
Wear long-sleeved shirt	1	Ref	0.16 (−0.02, 0.33)	0.23 (0.03, 0.42)	0.08
	2	Ref	0.02 (−0.15, 0.20)	0.05 (−0.15, 0.25)	0.88
Wear sunglasses	1	Ref	0.11 (−0.04, 0.26)	0.18 (0.01, 0.35)	0.12
	2	Ref	−0.10 (−0.25, 0.05)	0.05 (−0.12, 0.22)	0.08
Wear sunscreen	1	Ref	−0.01 (−0.19, 0.16)	0.18 (−0.02, 0.38)	0.06
	2	Ref	−0.19 (−0.37, −0.01)	−0.11 (−0.32, 0.09)	0.12
Intentional tanning frequency; 1_never_–5_always_	1	Ref	0.11 (0.00, 0.22)	0.00 (−0.12, 0.13)	0.04
	2	Ref	0.00 (−0.11, 0.11)	0.02 (−0.14, 0.10)	0.91
			Relative risk (95% CI)	Relative risk (95% CI)	
Sunburn	1	Ref	1.15 (0.58, 2.29)	1.13 (0.52, 2.48)	0.92
	2	Ref	1.89 (0.86, 4.19)	1.97 (0.84, 4.65)	0.26
Whole-body skin examination	1	Ref	0.95 (0.63, 1.42)	0.87 (0.54, 1.40)	0.84
	2	Ref	1.03 (0.81, 1.30)	1.19 (0.93, 1.53)	0.26
Psychological outcomes			Mean difference (95% CI)	Mean difference (95% CI)	
Melanoma-related worry; 1_less_–5_more_	1	Ref	−0.02 (−0.13, 0.08)	0.11 (−0.01, 0.23)	0.03
	2	Ref	0.04 (−0.07, 0.14)	0.18 (0.05, 0.30)	0.007
Psychological distress & well-being; 0_low_–100_high_	1	Ref	0.54 (−1.69, 2.78)	−2.70 (−5.25, −0.15)	0.008
	2	Ref	−1.41 (−3.66, 0.84)	−2.20 (−4.77, 0.36)	0.24

*CI* confidence interval, *SED*standard erythemal dose.

^a^Adjusted for baseline measurements, randomization stratification variables (sex, age group, state/territory of residence), and risk group by follow-up interaction.

^b^Refers to number at follow-up 1/follow-up 2, based on Sun Protection Index total score.

^c^*P* value for UV exposure (SEDs/day) differences between genomic risk groups used an analysis of covariance (ANCOVA). *P* value for differences between genomic risk groups for all other variables used generalized linear mixed models (GLMMs) with random intercepts for continuous outcome measures, and generalized estimating equations (GEEs) with a log link function for binary outcome measures.

Further evaluation of the intervention effect stratifying by both genomic risk and traditional risk groups showed several differences across the risk groups, with individuals with both high traditional and high genomic risk having the greatest increase in sun protection behaviors and skin examinations (Supplementary Table 6 and text).

## DISCUSSION

Polygenic scores are increasingly of interest for population risk stratification for cancer screening and prevention [29, 30], yet few studies have evaluated their real-world clinical utility and public health impact in sufficiently powered clinical trials. This population-based trial evaluated the impact of providing personalized melanoma genomic risk information, based on a polygenic risk score, on sun-related prevention and early detection behaviors. Contrary to the null findings from a previous systematic review on the impact of communicating genetic risks for a range of diseases on risk-reducing health behaviors [8], our findings highlight potential opportunities for using a polygenic risk score for precision melanoma prevention and early detection in the general population.

The most pronounced behavioral effect of the intervention was on reducing sunburn incidence. Sunburns at any age are a well-established, strong risk factor for melanoma [31]; thus, the reduction in sunburn incidence observed in this study could have considerable impact on reducing melanoma incidence in the population.

A key finding was that the impact of the intervention on behavioral outcomes differed according to population subgroups. The stronger effects of the intervention for people with high traditional melanoma risk is important as the majority of melanomas in the population occur in this subgroup [32], thus corresponding to where most gains in prevention can be found. We also found that effect modification of the primary outcome by genetic determinism, with a protective effect evident among those perceiving weaker genetic determinism. Thus, addressing pre-existing deterministic views in the provision of personal genomic risk information to the population could facilitate behavior change. This finding may also be relevant for older people who may have fatalistic attitudes toward sun protection behaviors if they believe that “the damage is already done.” [1, 33] The stronger intervention effect on sun protection behaviors among participants aged 45–69 years may impact future melanoma incidence for this age group as sun protection measures such as sunscreen use have been shown to be effective in reducing melanoma incidence when used at any age [1]. The importance of skin cancer prevention at all ages was emphasized in our general educational booklet and is important to counter fatalistic attitudes. Stronger effects of the genomic risk intervention among women than men was also observed by Godino et al. for the effect of a type 2 diabetes genetic risk estimate on physical activity [34]. Women may also be more willing than men to engage in preventive strategies [35].

The fact that participants in both intervention and control arms reduced their UV exposure highlights the possibility that other study factors may have motivated reduced personal UV exposure in the study, such as the general educational booklet, participation in the trial itself, or the monitoring of behaviors (Hawthorne effect) [36]. Objectively measured UV exposure did not differ between intervention and control groups despite a reduction in sunburns and increase in sun protection behaviors, but this can be explained by participants being instructed to wear the dosimeter on the wrist and uncovered by clothing. Therefore, the dosimeter measurement would not have been affected by sun protection behaviors except for shade use.

The short-term benefits of the intervention on sun protection behaviors were attenuated by 12 months, suggesting regular reminders may be needed. Lessons from other skin cancer prevention interventions show that population gains in behavior can be quickly eroded without sustained investment [37]. Sustained effects at follow-up 2 were observed for sunburns and skin examinations—this might be facilitated by having sent a summary of participants’ personal genomic risk information to their GP.

Several concerns about providing personal genomic risk information to the healthy population have been identified. Encouraging cancer screening especially for people at low risk may increase the likelihood of overdiagnosis, which is associated with physical and psychological harms [38]. It is thought low-risk results might also provide false reassurance and encourage risky behaviors [38]. This concern was not borne out by our study, as prevention behaviors did not worsen in the low genomic risk group. A higher level of genomic risk was associated with greater sun protection behavior change, consistent with a systematic review on the behavioral impact of return of genetic test results for complex diseases [39].

Consistent with other studies [8, 39], there were no adverse impacts of the intervention on psychological outcomes in our study, and no increase in melanoma-related worry even for high-risk groups. The MICRA assessment showed participants at high genomic risk had consistently higher distress and uncertainty scores than those at low risk, but overall the scores were low [40]. Interestingly, the high genomic risk group also had higher scores on the positive experiences subscale, which includes participants’ satisfaction with family support and communication during genetic testing.

Our study had several strengths. It was population-based, nationwide and adequately powered. Loss to follow-up was minimal and equal between groups, thus minimizing selection bias. The primary outcome was objectively measured and we achieved excellent wear adherence. The other outcomes in our study were self-reported, which could lead to reporting bias; however we used validated measures wherever possible.

The consent rate was low (3.4% overall) and could be a concern for generalizability of the results. The true consent rate would be higher after accounting for the oversampling of younger people and men (e.g., consent was 7% for women aged 45–69 years), people with an outdated mailing address in the Medicare database (usually ~3%) or who did not return the consent form because they were ineligible to participate (e.g., due to ancestry or a previous melanoma, estimated at ~20%). Other population-based research studies recruiting through the Medicare database have experienced consent rates less than 10% [41]. Our pilot study recruited participants from a database of people interested in cancer research and had 41% consent [42]. Large-scale implementation of a population-based approach to providing genomic risk information would likely have higher acceptability and uptake if conducted in primary care practice, where doctors anticipate delivering this type of information in the future [43]. It could also be delivered by nurses or educators trained in genomics [44]. A previous study of *MC1R* gene testing in a diverse primary care population in the United States showed 33% uptake of testing [11]. Another study showed that up to 40% of the population would rather not know their chances of getting cancer [45].

Compared to Australian population data (Australian Bureau of Statistics), participants in our study were more likely to have been born in Australia (78% versus 70%), have higher education levels (45% with a university degree versus 28%), and have higher residence-based socioeconomic indicators (mean socio-economic indexes for areas [SEIFA] [46] score 1,019 for those randomized versus 1,005 for those invited). They were also more likely to have a family history of melanoma (19% versus <6% in other studies) [47]. Our findings are also limited to populations with European ancestry. It is essential that genomic databases include more diverse populations and that any potential benefits of genomics research be applied equitably across different populations subgroups.

In conclusion, we demonstrated that personal melanoma genomic risk information did not influence objectively measured patterns of sun exposure but had beneficial impacts on sun protection, sunburn and skin examinations, which varied by population subgroup, without evidence of psychological harm. These findings will inform a cost-effectiveness analysis, and have policy and clinical relevance regarding the potential use of genomic risk information for precision cancer prevention and early detection strategies at the population level.

## Supplementary information

Supplementary Materials

## Data Availability

Participants have not given consent for their de-identified data to be deposited in a repository but they have indicated whether they consent to their data and sample being used for other research purposes that are ethically approved. Requests for obtaining de-identified data should be addressed to the corresponding author. The Study Protocol and Statistical Analysis Plan are available and have been published elsewhere [18, 19].
