# Supplementary material for: Impact of personal genomic risk information on melanoma prevention behaviors and psychological outcomes: a randomized controlled trial
Source: Genet Med. 2021 Aug 12;23(12):2394–403. doi: 10.1038/s41436-021-01292-w (PMC8629758; doi:10.1038/s41436-021-01292-w)
Supplement: Supplementary file 1 — Supplementary Materials [file 41436_2021_1292_MOESM1_ESM.pdf]

Supplementary file

Contents

SUPPLEMENTARY MATERIALS AND METHODS 2
SUPPLEMENTARY RESULTS 3
SUPPLEMENTARY REFERENCES 5
Supplementary Figure 1. 6
Supplementary Figure 2. 7
Supplementary Figure 3. 8
Supplementary Table 1. Baseline participant characteristics by intervention arm, stratified by traditional risk groups 9
Supplementary Table 2. Satisfaction, understanding and uptake of the intervention, for participants in the intervention arm stratified by genomic risk group 10
Supplementary Table 3. Intervention participants' recall of their personal genomic risk category .. 11
Supplementary Table 4. Descriptive data at baseline and follow-up for primary and secondary outcomes 12
Supplementary Table 5. Multidimensional Impact of Cancer Risk Assessment (MICRA) scores, stratified by genomic risk groups 13
Supplementary Table 6. Outcomes in the intervention arm only, stratified by genomic risk and traditional risk categories 14

Genetic risk booklet........15
General educational booklet........27

SUPPLEMENTARY MATERIALS AND METHODS

Participants

All Australian citizens and other eligible residents (including permanent residents and New Zealand citizens) are registered with Medicare, which is Australia's publicly funded universal healthcare system. To ensure roughly equal numbers of participants by gender and age-group we oversampled younger people, especially younger men, as our pilot study indicated they had lower response rates.[1] Information about ancestry and previous melanoma was sought on the study consent form.

Statistical analysis

The primary analysis compared the intervention (genomic risk) and control (usual care) arms for mean differences in UV exposure measured as log-transformed daily SEDs at 12-months, using an analysis of covariance (ANCOVA) adjusted for baseline values and randomised stratification factors (gender, state of residence, age-group), and presented separately for traditional risk groups (high, low). Where outcomes were assessed at three time points (baseline, follow-up 1 and follow-up 2) data were analysed using generalised linear mixed models (GLMMs) with random intercepts for continuous outcome measures, and generalised estimating equations (GEEs) with a log link function for binary outcome measures to estimate relative risks and 95% confidence intervals (CIs).

Subgroup analyses assessed differences in intervention effects (comparing intervention versus control arms) across pre-specified subgroups,[2] including sex, age (18-44, 45-69 years), state or territory of residence, health literacy and numeracy,[3] family history of melanoma, personal history of keratinocyte cancer, education, socioeconomic status, children, medications, and attitudes towards genetic determinism (i.e. views on the extent to which genetic make-up determines whether or not a person will develop melanoma[4]). Based on the literature and expertise of the research team, it was hypothesized that the effect of the intervention on behaviour change or psycho-social

outcomes may be influenced (moderated) by these subgroup factors. Tests of effect modification were performed by fitting the interaction and main effects for the intervention group and relevant subgroup into the models adjusted for baseline scores.

Analyses were performed using complete case analysis as there were <1% missing values for the completed measures.[5] Analyses were conducted using SAS version 9.4 and figures were created using R 3.6.1 forestplot and plotrix R packages.[6,7]

SUPPLEMENTARY RESULTS

Intervention process measures

Uptake of the intervention

Only four participants assigned to the intervention arm did not provide a saliva sample and thus did not receive the intervention. On average, intervention participants read most of their personalised genomic risk booklet (Supplementary Table 2) and this was similar across genomic risk groups.

Recall of personal genomic risk category

Intervention participants' correct recall of their personal genomic risk category was 98%, 67% and 92% for the low/average/high groups, respectively, at follow-up 1, and 95%, 55% and 83%, respectively, at follow-up 2 (Supplementary Table 3). Of intervention participants in the average genomic risk category who inaccurately recalled their result, they were more like to recall a lower than average risk result than a higher than average risk (Supplementary Table 3).

Intervention materials - understanding and satisfaction

On average, intervention participants found the presentation of their personal genomic risk information moderately to very easy to understand (Supplementary Table 2). There was very high satisfaction with the genetic counsellor call and personalised genomic risk booklet (Supplementary Table 2). Understanding and satisfaction were rated slightly higher by participants in the low

genomic risk category compared to those in the average or high genomic risk categories. Scores reported at follow-up 2 were similar to those reported in follow-up 1.

General educational booklets - satisfaction and amount read (intervention and control arms)

At follow-up 1, all participants were asked how much of the general educational booklet they read, on a scale of 1 to 5 (1=from cover to cover, 5=not read); the mean score was 1.9 (SD 1.0) for intervention participants and 2.2 (SD 1.2) for control participants ($P<0.001$ for difference between trial arms). On a scale of 1 to 10 (1=not at all satisfied and 10=extremely satisfied), satisfaction with the general educational booklet on melanoma prevention and early detection measured at follow-up 1 was 8.6 (SD 1.6) for intervention participants and 7.5 (SD 2.1) for control participants ($P<0.001$ for difference between trial arms). Similar scores were given at follow-up 2.

Secondary outcomes

Evaluation of the intervention effect stratifying by both genomic risk and traditional risk groups

Evaluation of the intervention effect stratifying by both genomic risk and traditional risk groups, with low traditional risk and low/average genomic risk as the reference category, showed several differences across the risk groups (Supplementary Table 6). Individuals with both high traditional and high genomic risk had the greatest increase in sun protection behaviours (total score, hat wear, sunscreen use) at follow-up 1, and 46% higher incidence of skin examinations at follow-up 2. Melanoma-related worry was reduced in all risk groups except the high/high risk group where the scores remained constant (2.02, 2.04, 1.96 over the 3 time-points). For the discordant traditional/ genomic risk groups, there were improved sun-protection behaviours (total score) at

follow-up 1, and the high traditional risk/low-average genomic risk group had 35% higher incidence of skin examinations at follow-up 2.

SUPPLEMENTARY REFERENCES

1. Smit AK, Espinoza D, Newson AJ, et al. A Pilot Randomized Controlled Trial of the Feasibility, Acceptability, and Impact of Giving Information on Personalized Genomic Risk of Melanoma to the Public. Cancer Epidemiol Biomarkers Prev. 2017;26(2):212-221.
2. Lo SN, Smit AK, Espinoza D, Cust AE, Managing Your Risk Study G. The Melanoma Genomics Managing Your Risk Study randomised controlled trial: statistical analysis plan. Trials. 2020;21(1):594.
3. Smith SK, Simpson JM, Trevena LJ, McCaffery KJ. Factors Associated with Informed Decisions and Participation in Bowel Cancer Screening among Adults with Lower Education and Literacy. Med Decis Making. 2014;34(6):756-772.
4. Hay J, Kaphingst KA, Baser R, Li Y, Hensley-Alford S, McBride CM. Skin cancer concerns and genetic risk information-seeking in primary care. Public Health Genomics. 2012;15(2):57-72.
5. Jakobsen JC, Gluud C, Wetterslev J, Winkel P. When and how should multiple imputation be used for handling missing data in randomised clinical trials - a practical guide with flowcharts. BMC Med Res Methodol. 2017;17(1):162.
6. Gordon M, Lumley T. Advanced Forest Plot Using 'grid' Graphics. 2016.
7. Lemon J. Plotrix: A package in the red light district of R. R-News. 2006;6:8-12.

A.

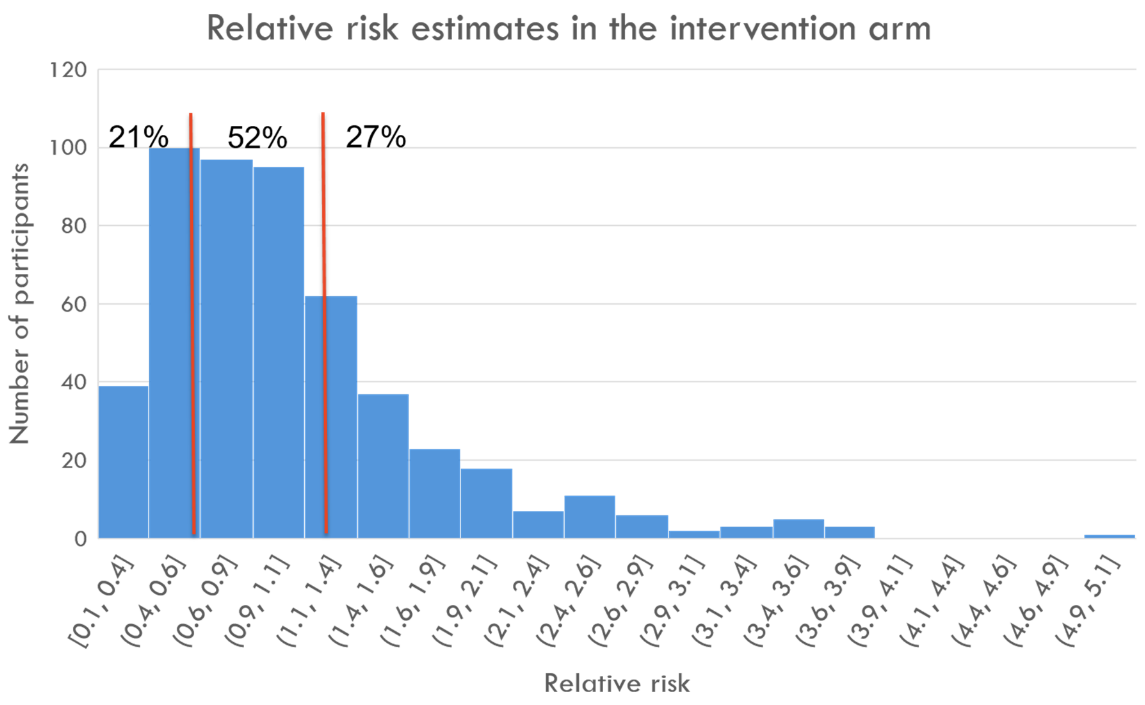

B.

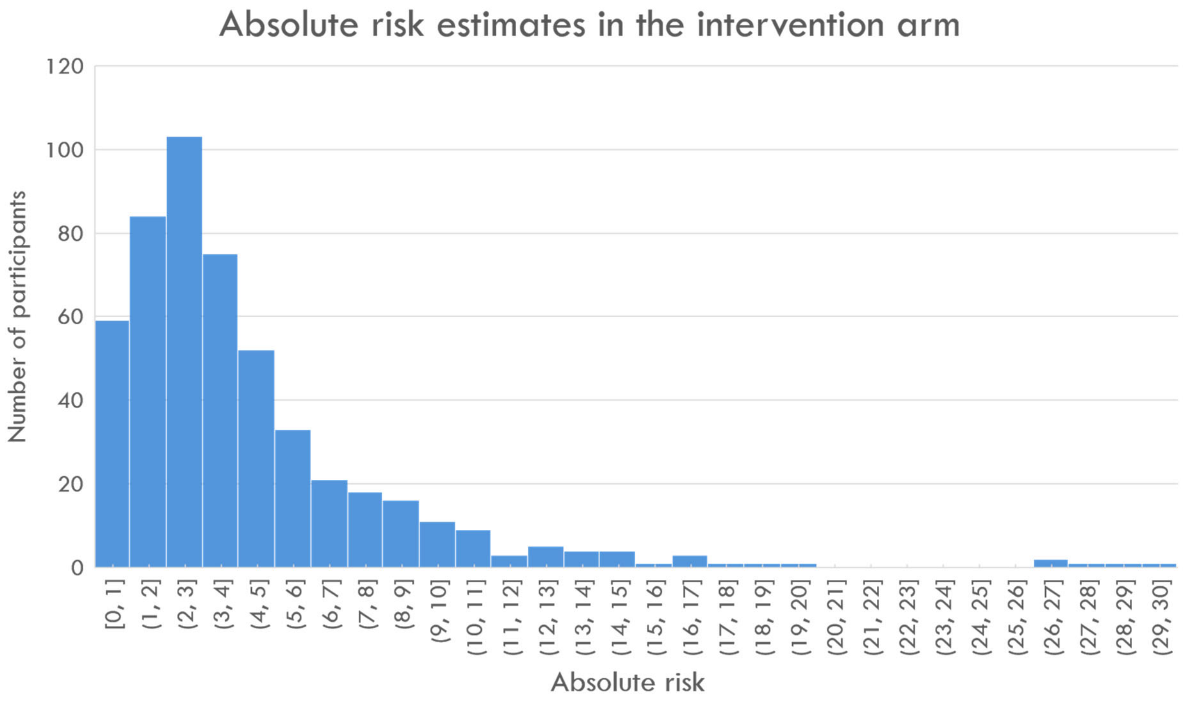

Supplementary Figure 1.

Distribution of personal genomic risk estimates for the intervention arm. A). Relative genomic risk estimates in the intervention arm overall, based on the polygenic risk score only. Red lines indicate cut points for the genomic risk categories: <0.56 = lower than average risk (n=21%), >=0.56 and <1.32 = average risk (n=52%), >1.32 = higher than average risk (n=27%). B). Absolute genomic risk estimates (percentage risk) in the intervention arm overall, based on the polygenic risk score and underlying age/sex/state-specific population melanoma incidence and competing mortality rates.

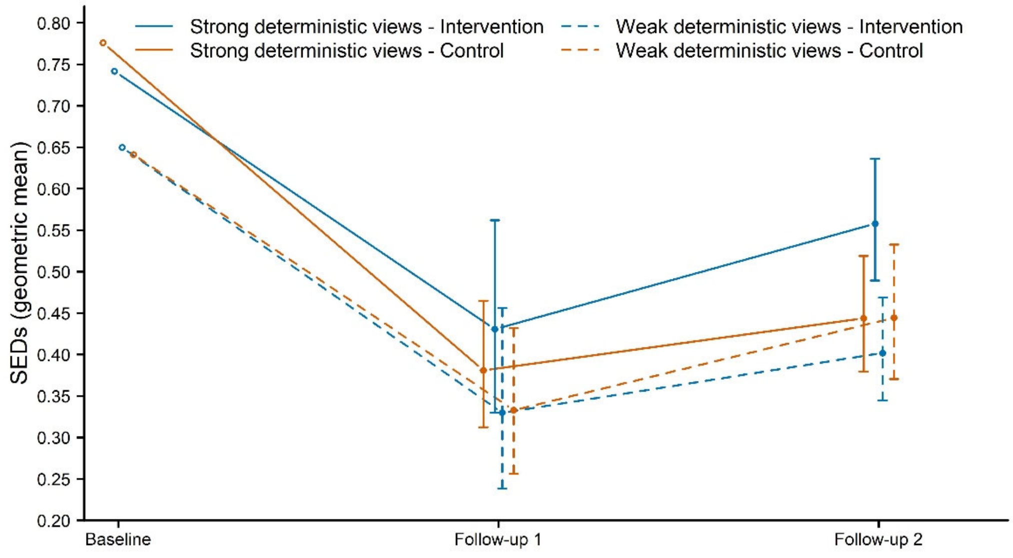

Supplementary Figure 2.

Objectively-measured daily Standard Erythemal Doses (SEDs) stratified by views on genetic determinism. Values are shown for baseline, follow-up 1 and 2 for intervention and control arms. Vertical bars represent 95% confidence interval (CI). At follow-up 2, the percentage difference and 95% CI comparing intervention with control arms among those with weak deterministic views was -7.56% (95% CI: -12.75, -2.05, p=0.008) and 6.46% (95% CI: 0.48, 12.81, p=0.03) among those with strong deterministic views (P-interaction=0.0008). Genetic determinism was measured as ‘How much do you think genetic make-up, that is characteristics that are passed down from one generation to the next, determine whether or not a person will develop melanoma?’ on a 5-point Likert scale and categorized as 4 or 5 (completely/moderately) vs. 1, 2, or 3 (not at all, slightly, somewhat).

Melanoma related worry **Mental Health Index (MHI-5)**

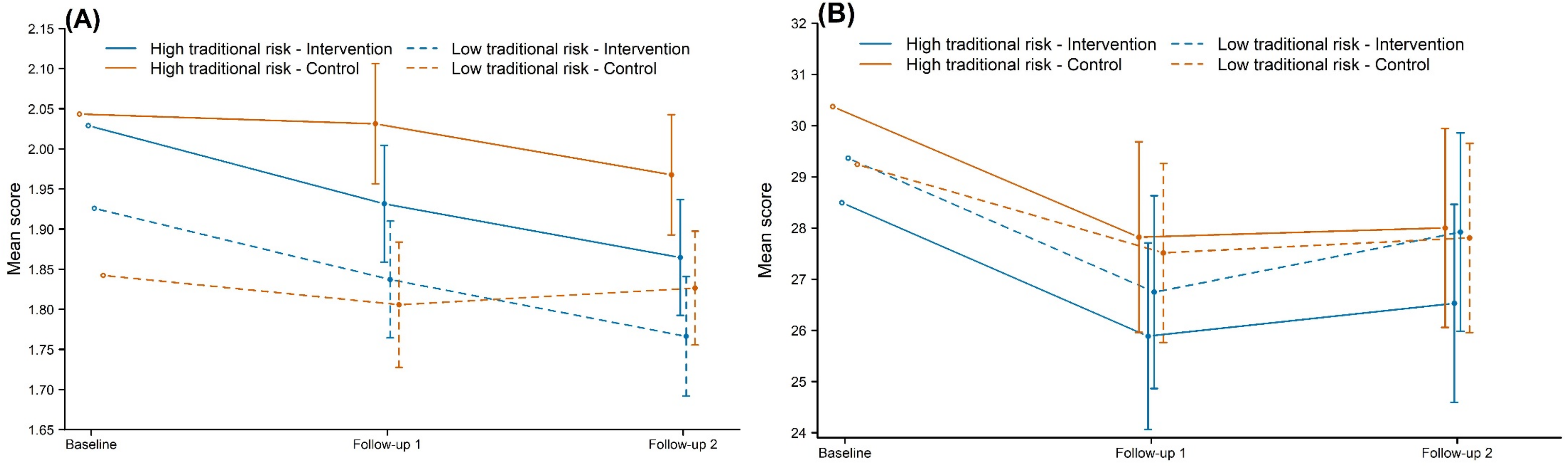

Supplementary Figure 3.

A. **Melanoma related worry** (mean score of 3 items on a Likert scale: 1=never, 5=always) at baseline, follow-up 1 and 2 in intervention and control arms, stratified by traditional risk groups. Vertical bars indicate 95% confidence intervals (CI). At follow-up 1, the mean difference comparing intervention with control arms in the low traditional risk group was -0.01 (95% CI: -0.09, 0.07; p=0.75) and -0.09 (95% CI: -0.17, -0.01; p=0.04) in the high traditional risk group. At follow-up 2, the mean differences were -0.10 (95% CI: -0.18, -0.01; p=0.02) and -0.09 (95% CI: -0.17, -0.01; p=0.04), respectively.

B. **Mental Health Index** (MHI-5; total mean score) at baseline, follow-up 1 and 2 in intervention and control arms, stratified by traditional risk groups. Vertical bars indicate 95% confidence intervals (CI). At follow-up 1, the mean difference comparing intervention with control arms in the low traditional risk group was -0.74 (-2.48, 0.99; p=0.40) and -0.74 (-2.61, 1.13; p=0.44) in the high traditional risk group. At follow-up 2, the mean differences were 0.32 (-1.44, 2.08; p=0.72) and -0.45 (-2.34, 1.44; p=0.64), respectively.

Supplementary Table 1. Baseline participant characteristics by intervention arm, stratified by traditional risk groups

	High traditional risk (n=505)		Low traditional risk (n=519[1])	
	Intervention (n=252)	Control (n=253)	Intervention (n=261)	Control (n=258[1])
	n (%)	*n (%)*	*N (%)*	*n (%)*
Country of birth				
Australia	209 (76.6%)	199 (78.7%)	200 (76.6%)	195 (75.6%)
New Zealand	9 (3.6%)	11 (4.4%)	11 (4.2%)	13 (5.0%)
United Kingdom	20 (7.9%)	21 (8.7%)	20 (7.7%)	19 (7.4%)
Other	14 (5.6%)	22 (8.7%)	30 (11.5%)	31 (12.0%)
Marital status				
Never married	45 (17.9%)	32 (12.7%)	54 (20.7%)	49 (19.0%)
Widowed	3 (1.2%)	0 (0%)	3 (1.2%)	6 (2.3%)
Separated or divorced	21 (8.3%)	10 (4.0%)	18 (6.9%)	14 (5.4%)
Married or in a de facto relationship	183 (72.6%)	211 (83.4%)	186 (71.3%)	189 (73.3%)
Highest level of education				
Primary school (or equivalent)	1 (0.4%)	4 (1.6%)	1 (0.4%)	2 (0.8%)
High school (or equivalent)	51 (20.2%)	54 (21.3%)	67 (25.7%)	59 (22.9%)
Certificate/diploma	79 (31.4%)	73 (28.9%)	97 (37.2%)	78 (30.2%)
University degree	121 (48.0%)	122 (48.2%)	96 (36.8%)	119 (46.1%)
Individual annual income before tax (AUD)				
Less than $50,000	83 (33.0%)	75 (29.6%)	89 (34.1%)	77 (29.8%)
$50,000-$99,999	81 (32.1%)	83 (32.8%)	98 (37.6%)	97 (37.6%)
$100,000-$149,999	44 (17.5%)	46 (18.2%)	36 (13.8%)	37 (14.3%)
$150,000-$199,999	13 (5.2%)	14 (5.5%)	14 (5.4%)	11 (4.3%)
$200,000 or more	18 (7.1%)	12 (4.7%)	8 (3.1%)	10 (3.9%)
Unsure/rather not answer	13 (5.2%)	23 (9.1%)	16 (6.1%)	26 (10.1%)
Current work status				
Working full time	118 (46.8%)	122 (48.2%)	125 (47.9%)	142 (55.0%)
Working part time/casual work	64 (25.4%)	54 (21.3%)	55 (21.1%)	53 (20.5%)
Student	8 (3.2%)	8 (3.2%)	16 (6.1%)	15 (5.8%)
Looking after home/family	11 (4.4%)	23 (9.1%)	20 (7.7%)	13 (5.0%)
Retired	42 (16.7%)	35 (13.8%)	33 (12.6%)	27 (10.5%)
Disabled/sick	3 (1.2%)	5 (2.0%)	6 (2.3%)	4 (1.6%)
Unemployed/looking for work	6 (2.4%)	6 (2.4%)	6 (2.3%)	4 (1.6%)
Previously diagnosed with non-melanoma skin cancer				
No	163 (64.7%)	154 (60.9%)	248 (95.0%)	239 (92.6%)
Yes	83 (33.0%)	90 (35.6%)	0 (0%)	3 (1.2%)
I don't know	6 (2.4%)	9 (3.6%)	13 (5.0%)	16 (6.2%)
If yes, type of skin cancer:[2]				
Basal cell carcinoma	76 (30.2%)	78 (30.8%)	0 (0%)	3 (1.2%)
Squamous cell carcinoma	25 (9.9%)	26 (10.3%)	0 (0%)	1 (0.4%)
Other	5 (2.0%)	5 (2.0%)	0 (0%)	0 (0%)
Previously diagnosed with other cancers (non-skin)				
No	232 (92.1%)	225 (88.9%)	244 (93.5%)	243 (94.2%)
Yes	17 (6.8%)	22 (8.7%)	16 (6.1%)	13 (5.0%)
I don’t know	3 (1.2%)	6 (2.4%)	1 (0.4%)	2 (0.8%)
First-degree blood relatives with a previous melanoma				
No	161 (63.9%)	153 (60.5%)	192 (73.6%)	191 (74.0%)
Yes	67 (26.6%)	73 (28.9%)	30 (11.5%)	27 (10.5%)
I don't know	24 (9.5%)	27 (10.7%)	39 (14.9%)	40 (15.5%)
First-degree blood relatives with previous non-melanoma skin cancer				
No	98 (38.9%)	102 (40.3%)	158 (60.5%)	147 (57.0%)
Yes	101 (40.1%)	79 (31.2%)	56 (21.5%)	52 (20.2%
I don't know	53 (21.0%)	72 (28.5%)	47 (18.0%)	59 (22.9%)
SEIFA (mean, standard deviation)[3]	1019.8 (62.1)	1021.1 (65.7)	1012.4 (65.8)	1022.0 (66.2)

[1] Excludes one participant who withdrew from the study
[2] Multiple responses possible
[3] Socio-Economic Indexes for Areas

Supplementary Table 2. Satisfaction, understanding and uptake of the intervention, for participants in the intervention arm stratified by genomic risk group

Intervention components (possible range)	Assessed at follow-up 1[1]			
	Low genomic risk (n=104)	Average genomic risk (n=264)	High genomic risk (n=127)	P-value
Amount read of booklet[2] (1-5)	1.75 (0.96)	1.77 (0.99)	1.77 (0.95)	0.89
Understanding of personalised risk booklet[3] (1-5)	4.76 (0.51)	4.51 (0.62)	4.34 (0.81)	<0.001
Satisfaction with genetic counsellor call[4] (1-10)	8.91 (1.44)	8.07 (2.20)	8.04 (2.54)	0.002
Satisfaction with personalised risk booklet[4] (1-10)	9.10 (1.24)	8.36 (1.65)	8.35 (1.70)	<0.001

[1] Data are shown as mean scores (standard deviation). The P-value is for the differences in the mean scores between genomic risk categories, adjusted for randomised stratification factors. There were no statistically significant differences in mean scores between follow-up 1 and follow-up 2, thus only follow-up 1 scores are shown.

[2] *How much of your genetic risk booklet did you read?* On a scale from 1 to 5: 1=from cover to cover, 2=most of it, 3=I only read the parts I felt were relevant to me, 4=briefly/skimmed, 5=I did not read the booklet.

[3] *In your genetic risk booklet, how easy or difficult was it to understand the presentation of your personal genetic risk of melanoma?* On a 5-point Likert scale: 1=very difficult, 2=moderately difficult, 3=neither easy nor difficult, 4=moderately easy, 5=very easy.

[4] *From 1 to 10 below, with 1 representing "Not at all satisfied" and 10 representing "Extremely satisfied" please select the number that best represents how satisfied you are with the information you have received in this study: Your personalised genetic risk booklet / Phone call from the Genetic Counsellor* On a scale from 1 to 10: 1= Not at all satisfied, 10=extremely satisfied.

Supplementary Table 3. Intervention participants' recall of their personal genomic risk category

Genomic risk category provided to participant at baseline	Recall by participant during follow-up N (%)		
	Lower than average	Average	Higher than average
		**Follow-up 1**	
Lower than average	102 (98)	2 (2)	0 (0)
Average	77 (29)	177 (67)	10 (4)
Higher than average	7 (6)	3 (2)	116 (92)
		**Follow-up 2**	
Lower than average	98 (95)	4 (4)	1 (1)
Average	102 (40)	139 (55)	14 (5)
Higher than average	8 (6)	13 (10)	104 (83)

As a measure of agreement, the Bowker Test of Symmetry was <0.0001 (Stat 82, DF 3) for follow-up 1 and <0.0001 (Statistic 96, DF 3) for follow-up 2.

Supplementary Table 4. Descriptive data at baseline and follow-up for primary and secondary outcomes

	**Baseline**				**Follow-up 1 (1-month post-intervention)**				**Follow-up 2 (12-months after baseline)**			
	**High traditional risk (n=505)**		**Low traditional risk (n=519)**		**High traditional risk (n=488)**		**Low traditional risk (n=506)**		**High traditional risk (n=487)**		**Low traditional risk (n=486)**	
	**Intervention (n=252)**	**Control (n=253)**	**Intervention (n=261)**	**Control (n=258)**	**Intervention (n=244)**	**Control (n=244)**	**Intervention (n=252)**	**Control (n=254)**	**Intervention (n=241)**	**Control (n=246)**	**Intervention (n=239)**	**Control (n=247)**
**Behaviours**												
Sun exposure – objectively measured												
Total daily SEDs[1] (GM, GSD)	0.74 (2.76)	0.70 (2.54)	0.66 (2.85)	0.72 (2.86)	0.41 (2.91)	0.39 (2.42)	0.34 (3.54)	0.33 (2.42)	0.47 (2.92)	0.44 (3.41)	0.48 (3.18)	0.45 (4.03)
Self-reported sun exposure[2]												
Total daily hours (SD)	2.81 (1.76)	2.52 (1.53)	2.62 (1.73)	2.95 (2.01)	2.56 (1.58)	2.62 (1.77)	2.64 (1.79)	2.80 (1.90)	2.69 (1.77)	2.57 (1.66)	2.56 (1.72)	2.66 (1.87)
Sun protection behaviours, mean (SD)[3]												
Total score	2.58 (0.50)	2.55 (0.54)	2.49 (0.52)	2.47 (0.54)	2.77 (0.53)	2.61 (0.53)	2.57 (0.55)	2.58 (0.59)	2.73 (0.51)	2.64 (0.50)	2.64 (0.52)	2.62 (0.57)
Limit peak sun exposure	2.54 (0.94)	2.54 (0.95)	2.51 (0.94)	2.36 (0.94)	2.73 (0.95)	2.70 (0.88)	2.65 (0.90)	2.50 (0.96)	2.69 (0.89)	2.61 (0.86)	2.73 (0.90)	2.57 (0.95)
Stay in shade	2.01 (0.77)	2.04 (0.75)	2.05 (0.80)	2.00 (0.80)	2.24 (0.79)	2.15 (0.78)	2.19 (0.83)	2.16 (0.82)	2.21 (0.79)	2.21 (0.77)	2.28 (0.79)	2.24 (0.84)
Wear a hat	2.54 (1.02)	2.50 (1.01)	2.38 (1.06)	2.45 (1.11)	2.80 (1.01)	2.58 (1.04)	2.47 (1.02)	2.62 (1.08)	2.70 (1.04)	2.64 (1.00)	2.57 (1.04)	2.57 (1.09)
Wear long-sleeved shirt	3.19 (0.88)	3.15 (0.97)	3.18 (0.90)	3.14 (0.94)	3.20 (0.87)	3.09 (0.95)	3.09 (0.93)	3.10 (0.98)	3.33 (0.83)	3.21 (0.91)	3.24 (0.89)	3.22 (0.92)
Wear sunglasses	3.08 (1.00)	2.99 (1.05)	2.85 (1.11)	2.95 (1.08)	3.23 (0.93)	3.07 (1.00)	2.94 (1.07)	3.02 (1.08)	3.16 (0.96)	3.00 (1.02)	2.93 (1.09)	2.99 (1.10)
Wear sunscreen	2.13 (0.95)	2.05 (0.93)	1.97 (0.94)	1.91 (0.99)	2.45 (1.02)	2.10 (1.00)	2.09 (1.02)	2.11 (1.04)	2.30 (1.05)	2.17 (0.90)	2.10 (0.98)	2.12 (0.98)
Intentional tanning,[3] mean (SD)	1.37 (0.76)	1.26 (0.60)	1.37 (0.68)	1.37 (0.73)	1.23 (0.51)	1.21 (0.52)	1.20 (0.54)	1.30 (0.72)	1.25 (0.58)	1.23 (0.61)	1.22 (0.54)	1.23 (0.61)
Sunburn (N,%)[3]	36 (14)	48 (19)	51 (20)	45 (17)	20 (8)	38 (16)	34 (13)	37 (15)	23 (10)	46 (19)	34 (14)	34 (14)
Skin-exams (N,%)	162 (64)	169 (67)	112 (43)	105 (41)	62 (25)	54 (22)	45 (18)	37 (15)	129 (54)	114 (48)	78 (32)	78 (32)
**Psychological outcomes**												
Mental Health Index, mean (SD)[4]	28.49 (14.77)	30.37 (15.82)	29.36 (15.81)	29.24 (15.18)	25.89 (14.46)	27.82 (14.77)	26.75 (15.17)	27.51 (14.16)	26.53 (15.18)	28.00 (15.22)	27.92 (15.36)	27.80 (14.71)
Melanoma-related worry, mean (SD)[4]	2.03 (0.66)	2.04 (0.57)	1.93 (0.63)	1.84 (0.64)	1.93 (0.58)	2.03 (0.59)	1.84 (0.59)	1.81 (0.63)	1.86 (0.57)	1.97 (0.59)	1.77 (0.59)	1.83 (0.56)

GM, Geometric mean; GSM, geometric standard deviation; SD, standard deviation; SED, standard erythemal dose

[1] At follow-up 1, UV dosimeters were worn by a subset of 238 participants (high traditional risk: intervention n=60, control n=57; low traditional risk: intervention n=60, control n=61). At follow-up [2,] UV dosimeters were worn by 948 participants (high traditional risk: intervention n=233, control n=234; low traditional risk: intervention n=238, control n=243).

[2] Missing data: baseline (n=1, intervention), follow-up 1 (n=1, intervention), follow-up 2 (n=1, control)

[3] Missing data: follow-up 2 (n=1, intervention)

[4] Missing data: follow-up 2 (n=4, intervention; n=2, control)

Supplementary Table 5. Multidimensional Impact of Cancer Risk Assessment (MICRA) scores, stratified by genomic risk groups

MICRA scores (possible range)	Follow-up 1					Follow-up 2				
	Overall (n=495)	Low genomic risk (n=104)	Average genomic risk (n=264)	High genomic risk (n=127)	P-value	Overall (n=482)	Low genomic risk (n=103)	Average genomic risk (n=254)	High genomic risk (n=125)	P-value
Total score (0-105)	13.22 (8.32)	9.18 (6.96)	12.47 (8.08)	18.10 (7.53)	<0.001	13.88 (8.14)	10.44 (7.77)	13.18 (7.58)	18.15 (7.81)	<0.001
Distress (0-30)	1.18 (2.70)	0.52 (1.93)	0.89 (2.28)	2.33 (3.59)	<0.001	1.07 (2.54)	0.40 (1.96)	0.84 (2.18)	2.10 (3.26)	<0.001
Uncertainty (0-45)	3.84 (4.72)	2.61 (3.89)	3.73 (4.84)	5.08 (4.84)	<0.001	3.70 (4.35)	2.80 (4.36)	3.58 (4.08)	4.69 (4.69)	0.007
Positive experiences (0-20)	8.20 (5.42)	6.06 (4.60)	7.85 (5.54)	10.69 (4.87)	<0.001	9.11 (5.77)	7.24 (5.41)	8.75 (5.89)	11.36 (5.08)	<0.001

Data are shown as mean scores (standard deviation). The P-value is for the differences in the mean scores between genomic risk categories, adjusted for randomised stratification factors.

Supplementary Table 6. Outcomes in the intervention arm only, stratified by genomic risk and traditional risk categories

	Follow-up 1 or 2	Intervention effect[1] (95% confidence interval) according to concordant and discordant genotype/traditional risk groups relative to the low traditional risk/low-average genomic risk group (reference category)				
		Low traditional risk Low/average genomic risk n=210/202[2]	Low traditional risk High genomic risk n=41/44[2]	High traditional risk Low/average genomic risk n=158/158[2]	High traditional risk High genomic risk n=86/82[2]	P-value[3]
**Behavioural outcome – objectively measured**			*% difference (95% CI)*	*% difference (95% CI)*	*% difference (95% CI)*	
UV exposure, SEDs/day	1	ref	-2.60 (-22.89, 23.04)	-3.37 (-17.72, 13.50)	-2.49 (-20.52, 19.62)	0.95
	2	ref	2.11 (-10.32, 16.25)	-2.07 (-9.98, 6.53)	-7.21 (-16.22, 2.77)	0.23
**Behavioural outcomes - self-reported**			*Mean difference (95% CI)*	*Mean difference (95% CI)*	*Mean difference (95% CI)*	
Sun exposure, total hours/day	1	ref	-0.02 (-0.43, 0.39)	-0.16 (-0.42, 0.09)	-0.18 (-0.49, 0.12)	0.52
	2	ref	-0.11 (-0.51, 0.29)	0.05 (-0.20, 0.31)	-0.16 (-0.47, 0.15)	0.58
Sun Protection Index; $1_{never/rarely}$-4_{always}	1	ref	0.13 (0.01, 0.26)	0.11 (0.04, 0.19)	0.18 (0.09, 0.28)	<0.001
Total score	2	ref	0.06 (-0.07, 0.18)	0.00 (-0.08, 0.08)	0.07 (-0.02, 0.17)	0.39
Limit midday sun exposure	1	ref	0.22 (-0.03, 0.47)	-0.01 (-0.17, 0.15)	0.19 (0.00, 0.38)	0.07
	2	ref	0.06 (-0.19, 0.31)	-0.13 (-0.28, 0.03)	-0.03 (-0.22, 0.16)	0.34
Stay in shade	1	ref	0.10 (-0.15, 0.35)	0.05 (-0.11, 0.20)	0.07 (-0.12, 0.25)	0.81
	2	ref	0.07 (-0.17, 0.31)	-0.10 (-0.25, 0.06)	0.03 (-0.16, 0.22)	0.41
Wear a hat	1	ref	-0.06 (-0.30, 0.17)	0.15 (0.00, 0.30)	0.24 (0.06, 0.42)	0.02
	2	ref	0.05 (-0.18, 0.28)	-0.03 (-0.18, 0.11)	0.14 (-0.05, 0.32)	0.33
Wear long-sleeved shirt	1	ref	0.08 (-0.18, 0.33)	0.08 (-0.08, 0.24)	0.18 (-0.01, 0.38)	0.30
	2	ref	-0.04 (-0.29, 0.21)	0.07 (-0.09, 0.23)	0.12 (-0.08, 0.32)	0.54
Wear sunglasses	1	ref	0.19 (-0.03, 0.41)	0.13 (0.00, 0.27)	0.15 (-0.02, 0.31)	0.10
	2	ref	0.13 (-0.09, 0.34)	0.05 (-0.08, 0.19)	0.15 (-0.02, 0.32)	0.29
Wear sunscreen	1	ref	0.20 (-0.06, 0.46)	0.22 (0.06, 0.38)	0.33 (0.13, 0.53)	0.004
	2	ref	-0.02 (-0.27, 0.24)	0.06 (-0.10, 0.22)	0.08 (-0.12, 0.28)	0.79
Intentional tanning frequency; 1_{never}-5_{always}	1	ref	-0.01 (-0.17, 0.14)	0.05 (-0.04, 0.15)	-0.07 (-0.19, 0.05)	0.24
	2	ref	0.09 (-0.06, 0.25)	0.06 (-0.04, 0.16)	-0.04 (-0.16, 0.08)	0.28
			Relative Risk (95% CI)	*Relative Risk (95% CI)*	*Relative Risk (95% CI)*	
Sunburn	1	ref	1.23 (0.57, 2.64)	0.69 (0.37, 1.28)	0.81 (0.41, 1.62)	0.54
	2	ref	1.77 (1.07, 2.93)	0.88 (0.50, 1.55)	0.94 (0.48, 1.83)	0.07
Whole-body skin examination	1	ref	0.93 (0.45, 1.92)	1.22 (0.83, 1.78)	1.03 (0.63, 1.67)	0.70
	2	ref	1.15 (0.77, 1.70)	1.35 (1.07, 1.71)	1.46 (1.12, 1.90)	0.03
**Psychological outcomes**			*Mean difference (95% CI)*	*Mean difference (95% CI)*	*Mean difference (95% CI)*	
Melanoma–related worry; 1_{less}–5_{more}	1	ref	0.05 (-0.10, 0.21)	-0.01 (-0.11, 0.08)	0.15 (0.03, 0.27)	0.05
	2	ref	0.14 (-0.01, 0.30)	0.01 (-0.09, 0.11)	0.16 (0.04, 0.28)	0.02
Psychological distress & well-being; 0_{low}-100_{high}	1	ref	-4.66 (-7.95,-1.37)	-0.25 (-2.29, 1.79)	-2.66 (-5.13, -0.20)	0.01
	2	ref	-0.36 (-3.60, 2.87)	-0.47 (-2.52, 1.59)	-2.04 (-4.56, 0.47)	0.46

[1] Adjusted for baseline measurements, randomisation stratification variables (sex, age group, state/territory of residence) and risk group by follow-up interaction.

[2] Refers to number at follow-up 1 / follow-up 2, based on sun protection index total score.

[3] P-value for UV exposure (SEDs/day) differences between risk groups used an analysis of covariance (ANCOVA). P-value for differences between risk groups for all other variables used generalised linear mixed models (GLMMs) with random intercepts for continuous outcome measures, and generalised estimating equations (GEEs) with a log link function for binary outcome measures.

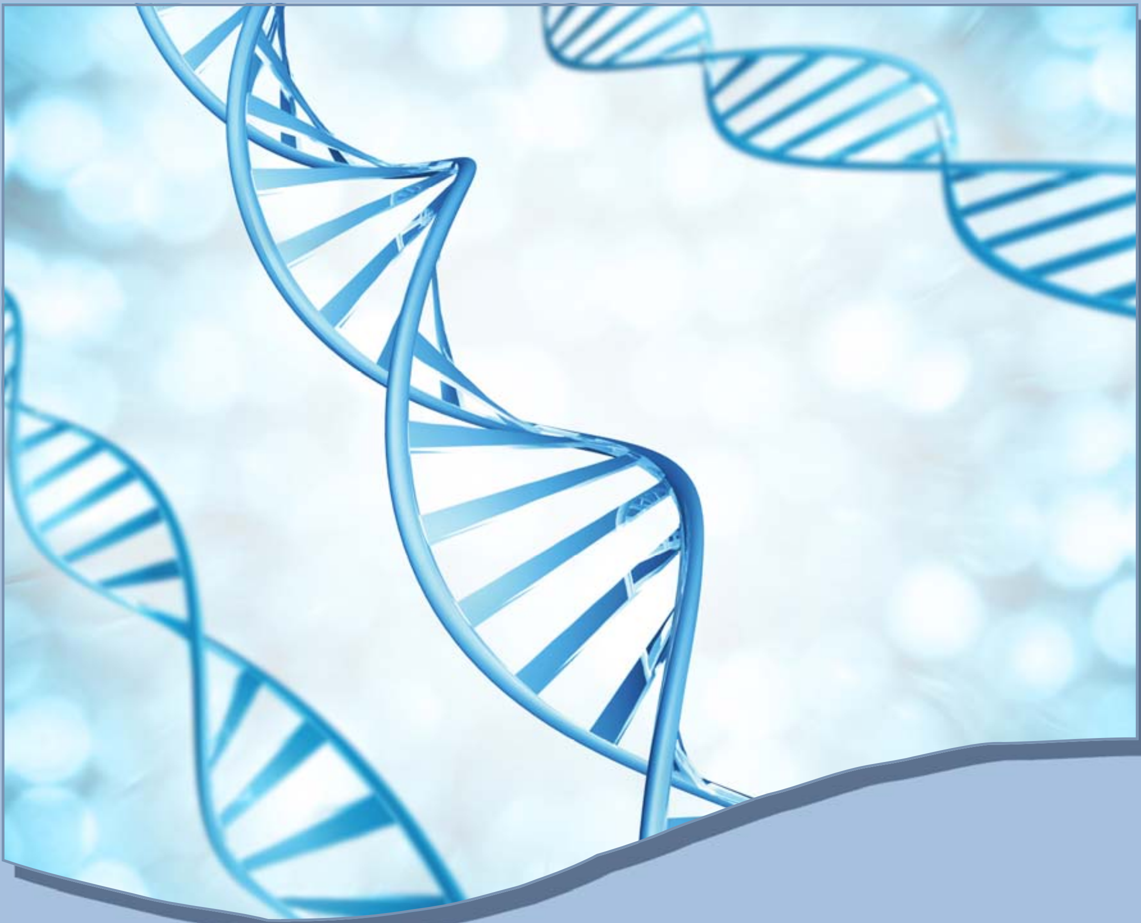

<Firstname>'s Genetic Risk of Melanoma

Now, let’s talk about it

Expect a call from the study genetic counsellor in the next few weeks. If you would like to speak to someone sooner, please call 1800 572 228 (free call) or email managing-risk.study@sydney.edu.au.

There’s more to it than genes

You CAN reduce and manage your risk (see page

Acknowledgements:

The study research team would like to acknowledge information obtained from other sources including GenoMEL (the international melanoma genetics consortium) and the PennSCAPE Study (Skin Cancer Awareness, Prevention and Education). The Managing Your Risk Study receives funding from The National Health and Medical Research Council. Some members of the research team have received funding for salary support from the National Health and Medical Research Council (NHMRC) and the Cancer Institute NSW.

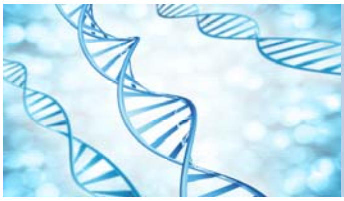

About this booklet:

This booklet gives your personal estimate of how likely you are to develop melanoma in the future, based on genetic testing. We call this an estimate of your remaining lifetime risk of melanoma. This booklet also describes how your remaining lifetime risk of melanoma was calculated, and what this risk means for you and your relatives.

For more information about how to manage your risk of melanoma, please refer to the separate booklet: “Melanoma Information: prevention and early detection” that was sent to you with this booklet.

If you have any questions about your risk information, please feel free to contact the study genetic counsellor on ph: 1800 572 228 (free call) or via email managing-risk.study@sydney.edu.au.

If you have any questions or concerns about your skin, your GP is the best person to contact in the first instance.

This study is based on research findings only and this type of genetic risk information is not yet available from your doctor or other health professional as part of standard health care.

Questions about the risk estimate

What is my remaining lifetime risk based on?

The estimate of your remaining lifetime risk of melanoma is based on your:

- **Age**
- **Gender**
- **Place of residence in Australia**
- **Genetic risk information from your DNA**

What is my genetic risk information?

Your genetic risk information was based on 20 different genes in your DNA that we know influence the risk of developing melanoma in people with European ancestry. These are genes in which natural variation is common in the population. These gene variations can be inherited and are thought to explain about 10-20% of new cases of melanoma in the population. Each gene change might have only a small influence on your melanoma risk, but depending on how many gene variations you have inherited, your risk could be quite high. Some of the genes include those that influence skin colour, number of moles, or skin sensitivity to the sun.

Your Remaining Lifetime Risk

Based on your genetic risk, you have a **higher than average risk** of melanoma. However, this does not take into account non-genetic risk factors such as past sunburns (see page 9).

Everyone is at risk of developing melanoma. Not everyone at higher than average risk will develop melanoma, but they have a higher chance than the average person. Even people at lower than average risk might develop melanoma.

Being aware of your risk is important, since it's the first step in taking precautions to reduce your risk.

Average Remaining Lifetime Risk

The average 64 year old man in NSW has a **5%** risk of developing melanoma during the rest of their life. In other words, 5 out of 100 men aged 64 living in NSW will develop melanoma during the rest of their life and 95 out of 100 will not.

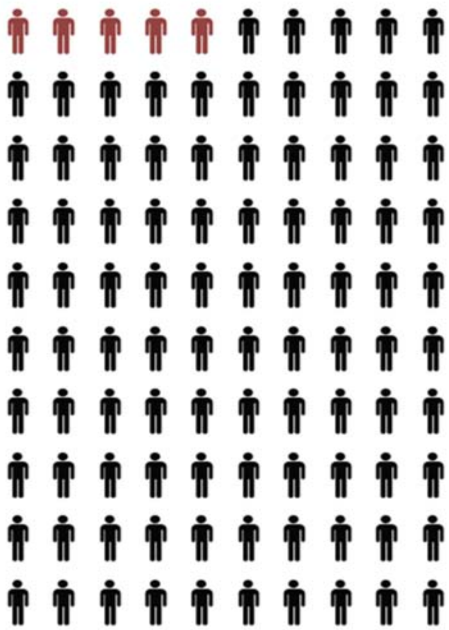

Your Remaining Lifetime Risk

Your personal risk of developing melanoma during the rest of your life is **9**%. This is based on genetic risk information from your DNA as well as the fact that you are aged 64, male and living in NSW. In other words, 9 out of every 100 men living in NSW who have the same age and genetic risk as you will develop melanoma during the rest of their life and 91 out of 100 will not.

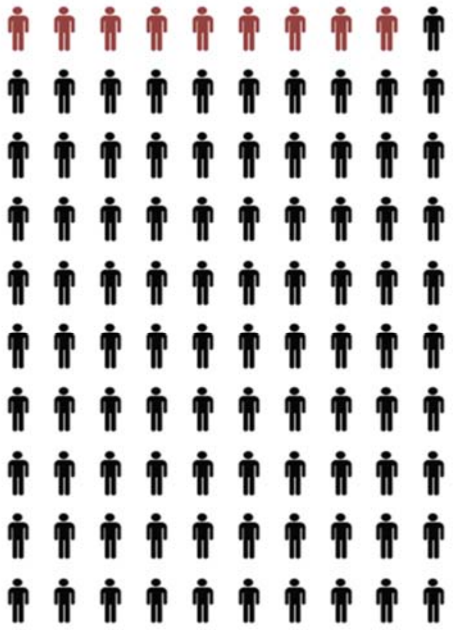

How accurate is the genetic risk estimate?

We have calculated your risk estimate based on the best available information. Some factors that influence the accuracy of your genetic risk estimate include:

1. Some uncertainty around the risk estimate

The risks for each gene variation that we used to calculate your lifetime genetic risk contain some uncertainty. Taking this uncertainty into account means that your actual genetic risk of melanoma could be higher or lower than the estimate shown in this booklet.

2. Your ancestry and where you have lived

Our information about these gene variations comes from studies of thousands of people around the world with European ancestry. Your lifetime melanoma risk is also based on where you currently live. If you do not have European ancestry, or if you have lived overseas or in different States of Australia, then your calculated lifetime risk of developing melanoma may not be accurate.

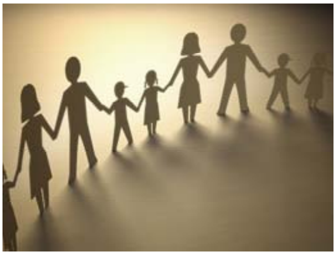

It is also possible that future research could change the meaning of the results we have given you. If you have questions in the future, please contact your GP or a genetic counsellor.

Does my lifetime risk estimate for melanoma include all my risk factors?

No. Your risk estimate is based on your genetic make-up, age, gender and where you currently live – it does not take into account other risk factors for melanoma. For example, your actual risk of melanoma may be higher than we estimated if you have:

- lots of moles,
- a family history of melanoma,
- had many sunburns,
- spent a lot of time in the sun especially as a child,
- used sunbeds, or
- immuno-suppression.

Your genetic risk information does not include all genes that are known to be associated with melanoma, as some genetic variations are very rare, difficult or costly to test.

If you have a strong family history of melanoma then you may be at high risk of melanoma regardless of the risk information shown from this study. Your GP may refer you to a family cancer clinic.

What does this information mean for my relatives?

Your relative's risk of melanoma may be similar to your risk, but not necessarily, as many different genes are involved. You can talk to the study genetic counsellor or your GP if you would like further information.

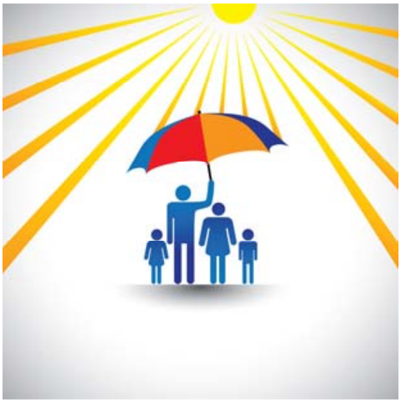

What can I do to reduce and manage my risk?

Your genetic variation won't change. But whatever your genetic risk level, the good news is that you can reduce your future risk of melanoma, whether you are 18 or 70, by being careful about when you spend time in the sun and by practising good sun protection behaviours. There are three things you can regularly do to reduce your risk of developing melanoma and increase the chances of detecting melanoma early:

1. Protect your skin from the sun

SLIP on sun protective clothing such as a long sleeved shirt
SLOP on SPF 30+ sunscreen and re-apply regularly
SLAP on a wide-brimmed hat
SEEK shade
SLIDE on sunglasses

This is especially important when the UV Index is 3 or above. .

2. Check your skin for melanoma

You should try to become familiar with your skin, and be alert for new or changing skin lesions and moles.

3. Have a health professional check your skin for melanoma

GPs and dermatologists use a number of tools and techniques to examine skin thoroughly. It is important to get a professional skin check by a doctor if anything suspicious appears on your skin.

See separate booklet **"Melanoma Information: prevention and early detection"**, for other practical information.

Who can I talk to about my risk?

A genetic counsellor: You can talk to the study genetic counsellor if you are concerned or have any questions about your risk.

Your GP: On the study consent form when you enrolled, you may have chosen for a copy of your risk information to be sent to your GP. Your GP is able to discuss with you any concerns about your skin. They may also refer you to a dermatologist.

What is a genetic counsellor?

A genetic counsellor is someone who is able to provide information to individuals and families about genetic conditions including certain types of cancer. They organise and assist with genetic testing, and discuss genetic test results and risk information. Genetic counsellors provide options and support for the decision-making process, both before and after genetic testing. They also help to communicate information within families about the impact of genetic test results.

How can I contact a genetic counsellor?

The genetic counsellor in this study is available for you to discuss your risk information. They will be able to explain the implications of your risk information and can give you advice on how to manage and reduce your risk of melanoma. If you want to speak with the study genetic counsellor please call 1800 1800 572 228 (free call) or email managing-risk.study@sydney.edu.au.

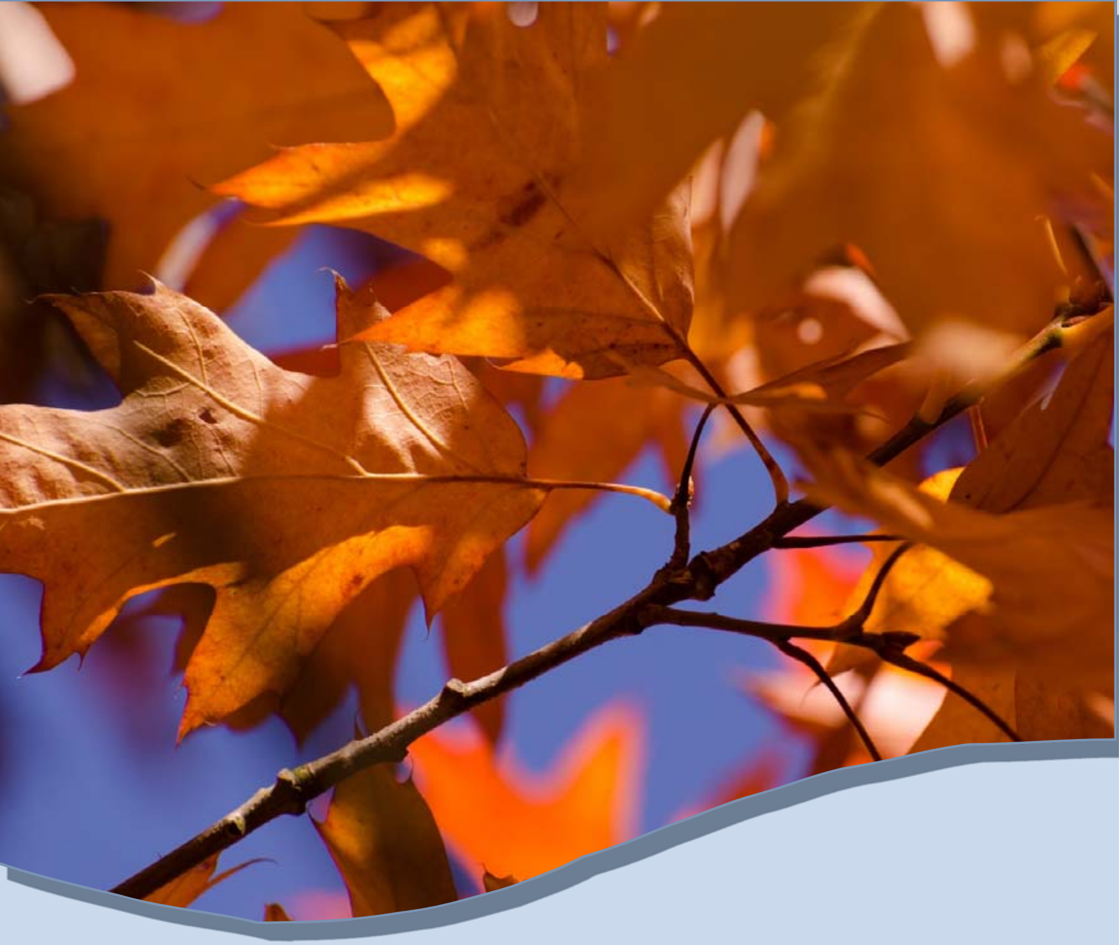

Melanoma Information:

prevention and early detection

Know Your Risk

Be Sun Smart - Protect your skin from the sun

Plan for Change, Starting Now

Contents

What is melanoma? 4

What causes melanoma? 5

How can I reduce my risk of melanoma? 7

1. Protect your skin from the sun..8

2. Check your skin for melanoma..12

3. Have a general practitioner or dermatologist check your skin for melanoma..14

How much sun do I need to maintain adequate Vitamin D levels?16

Other resources 17

About this booklet

This booklet provides you with information about melanoma and its risk factors. It also gives tips for spending time in the sun, using sun protection and checking your skin, which can help to reduce your risk of developing melanoma and lead to early detection. At the back of the booklet there is a list of resources and organisations that you can contact for free to get more information about melanoma and other skin cancers. If you have further questions or concerns about melanoma, please see your GP to discuss.

What is melanoma?

Melanoma is a form of cancer that develops in the skin's pigment cells (melanocytes).

Melanocytes produce melanin to help protect the skin from ultraviolet (UV) radiation i.e. sunlight. When these cells clump together in the skin during childhood or adolescence they form a mole.

Most moles are harmless, but sometimes the melanocytes in a mole can grow and divide in an uncontrolled way, resulting in a melanoma. A melanoma can develop anywhere on the skin, not just from moles. About half of all melanomas arise from existing moles and half do not.

Melanoma is the most serious form of skin cancer and can grow very quickly if left untreated. It can spread to the lower part of your skin (dermis), enter the lymphatic system or bloodstream and then spread to other parts of the body e.g. lungs, liver, brain or bone. Fortunately, in most cases, melanoma does not spread to other parts of the body and the prognosis is good.

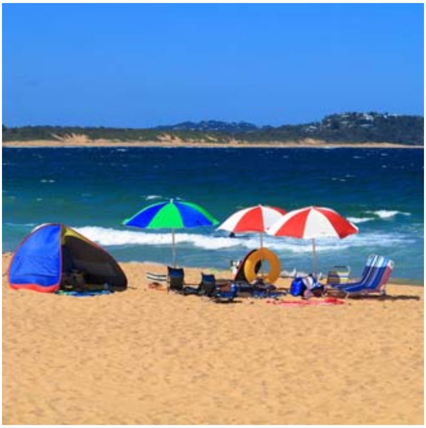

Australia has the highest incidence of melanoma in the world.

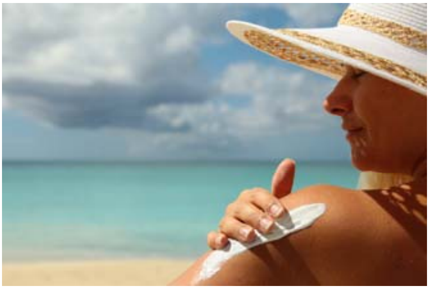

More than 12,500 Australians are diagnosed with melanoma each year and it is the most common cancer in young adults aged 15 to 44 years.

What causes melanoma?

While we know that some groups of people are at higher risk of developing melanoma than others, we cannot tell exactly who will develop melanoma. Understanding the factors that contribute to your risk is an important step towards reducing your chances of developing melanoma.

These factors can increase your risk of melanoma:

- **Too much** sun exposure, sunburn or using a solarium tanning machine, especially during childhood and adolescence
- Having **lots of moles**
- Having **other types of skin cancer**, for example basal cell carcinoma (BCC) and squamous cell carcinoma (SCC)
- **Fair** skin, **red** hair, **blue** eyes, or skin that **burns easily**
- Older age: like most cancers, the risk of developing melanoma **increases with age**. Melanoma is most common in people aged over 50. While younger people are less likely to get cancer, in those that do, melanoma skin cancer is the most commonly diagnosed cancer for people aged between 15 and 44
- **Family history**: relatives in your family who have had melanoma, especially if they developed it at a young age
- **Immuno-suppression**
- **Genetic** factors: some gene variations can be inherited in families

Other skin cancers

There are three main types of skin cancer: basal cell carcinoma (BCC), squamous cell carcinoma (SCC) and melanoma.

BCC is the most common form of skin cancer. This type of skin cancer rarely spreads to other parts of the body and typically develops on parts of the body that have been exposed to the sun.

SCC is the second most common form of skin cancer and is most frequently seen on sun-exposed areas, such as the head, neck, and back of the hands but it is possible to get it on any part of the body, including the inside of the mouth, lips, and genitals.

Melanoma is rarer than SCC and BCC, and is the most serious form of skin cancer. Early diagnosis is associated with better outcomes.

How can I reduce my risk of melanoma?

There are three things you can regularly do to reduce your risk of developing melanoma and increase the chances of detecting melanoma early:

1. Protect your skin from the sun

2. Check your skin for melanoma

3. Have a health professional check your skin for melanoma.

Whatever risk factors you already have or whatever you have done in the past with respect to your time in the sun, **the good news is you can reduce your future risk of melanoma at any age, whether you are 18 or 70, by being careful about how and when you spend time in the sun.**

Everyone changes at their own pace. There's no time like the present to begin!

1 Protect your skin from the sun

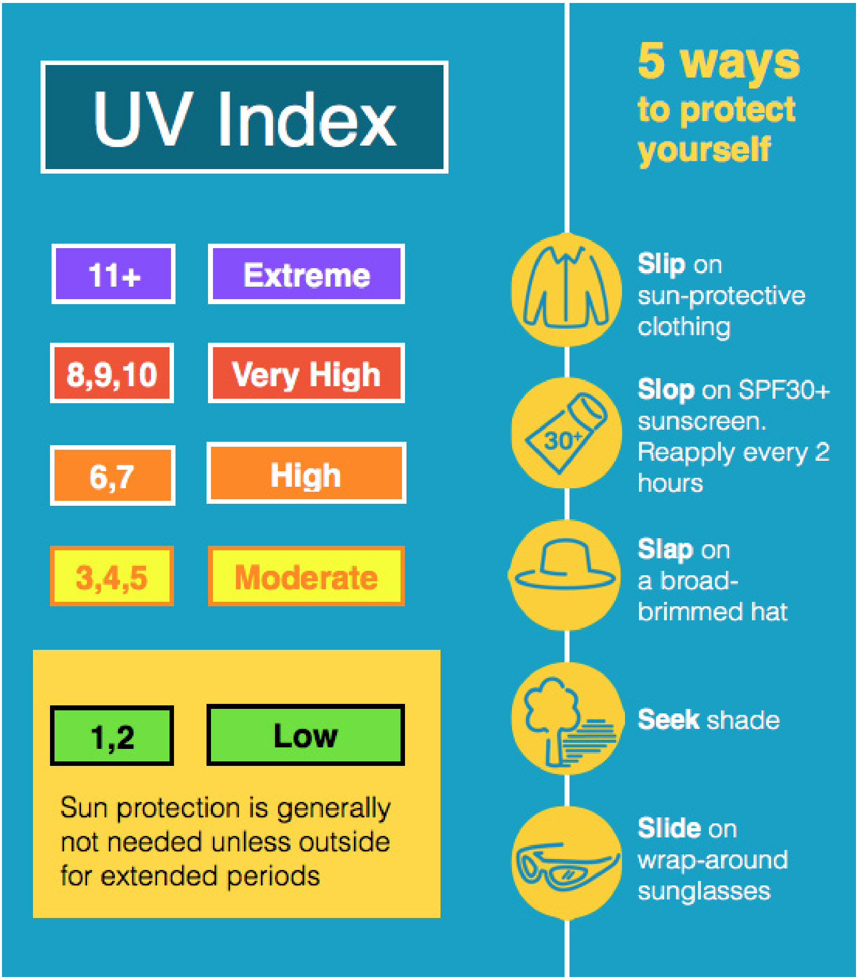

Planning is the key to successful change, and preventing melanoma takes planning too. When in the sun and the **UV Index is 3 or above**, it is important to remember to protect yourself in the following 5 ways:

- **SLIP** on sun protective clothing such as a long sleeved shirt
- **SLOP** on SPF 30+ sunscreen and reapply at least every two hours. Apply sunscreen liberally. Apply at least one teaspoon for the face, neck and ears, one teaspoon for each arm, two teaspoons for each leg, and two teaspoons for the torso. Most people don't apply enough sunscreen resulting in less protection than stated on the product.
- **SLAP** on a wide-brimmed hat
- **SEEK** shade
- **SLIDE** on sunglasses

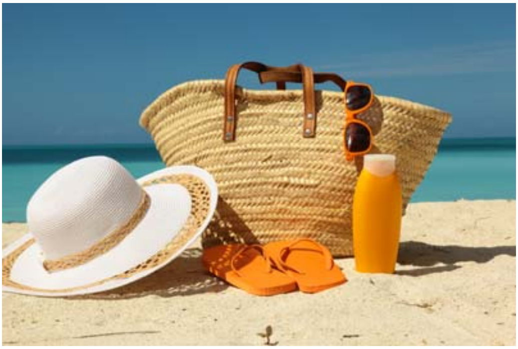

It is recommended that people use sun protection when the UV index is 3 or above

The UV Index is a simple way of describing the intensity of UV (ultraviolet) radiation from the sun at different times of the day. **The higher the UV Index value, the greater the potential for damage to your skin.**

The UV index and the times that sun protection is required are shown in the weather section of your daily newspaper, on the ARPANSA website at www.arpansa.gov.au/uvindex/realtime/index.cfm or using the free **SunSmart** app (see details below).

Download the App!

The free SunSmart mobile phone app contains useful information for any location in Australia. It can be downloaded for your tablet or mobile device from the App Store or Google Play Store.

The App includes information about the times each day when you should use sun protection for your location, as well as personalised alerts to remind you when sun protection is needed.

The App also includes a Vitamin D tracker to help you find out if you are getting enough sun exposure for Vitamin D, and a sunscreen calculator to help you work out if you are using enough sunscreen.

www.sunsmart.com.au/app

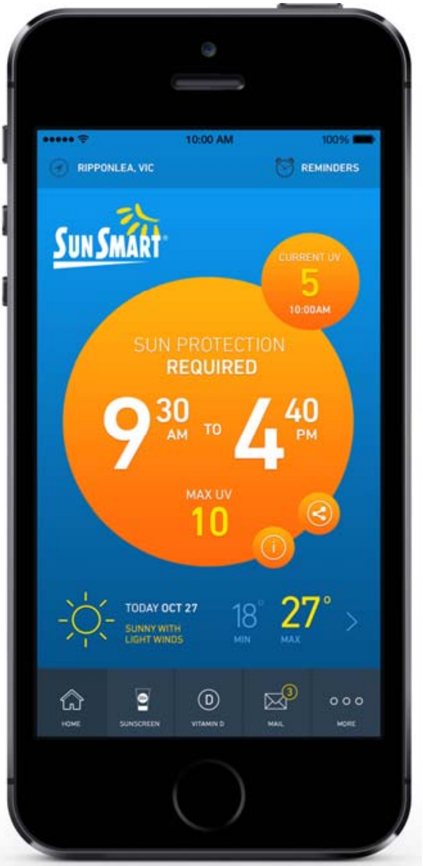

The UV index is higher in spring, summer and autumn than in winter, and is higher in the north than in the south of Australia. The UV index can be high even on cool and overcast days, so **you can't rely on clear skies or hot weather** to determine when you need to protect yourself from the sun. **Even in Spring, Australians often get large doses of UV**.

In the northern states of Australia, sun protection is needed all year around at certain times of the day. In these areas, it is better to spend time outside in the early morning or late afternoon.

People in southern states may not need sun protection in winter when the UV Index is likely to be below 3. However, sun protection is always needed at high altitudes or near highly reflective surfaces like snow and water.

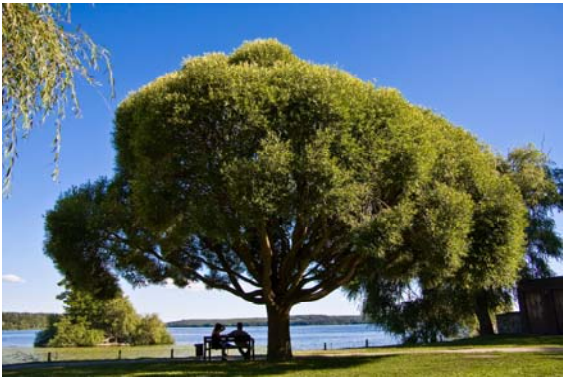

Try to avoid going out in the sun when the UV levels are at their peak (towards the middle of the day). If you do go outside when the UV Index is 3 or above, remember to use sun protection and to seek shade.

2 Check your skin for melanoma

You can check your own skin to ensure that if melanoma does develop, it can be detected at an early stage (when treatment is most successful). More than half of all melanomas are first discovered by the patient. You should try to become familiar with your skin, and be alert for new or changing skin lesions and moles.

What are moles?

Moles are harmless growths of pigment cells (melanocytes) in the skin. Almost everyone has moles and they appear on our body in our first 40 years of life. Moles can be flat or raised and can differ greatly in colour, size and shape.

Does the number of moles I have matter?

The more moles you have, the greater your risk of developing melanoma.

Do all melanomas start as moles?

About half of all melanomas arise from existing moles and half do not.

Should I have all my moles removed to prevent melanoma?

No. Clinical practice guidelines do not recommend removing non-suspicious moles because there is no evidence that this reduces your risk of developing melanoma or improves your health outcomes. There is also an increased risk of scarring and other complications with mole removal.

Did you know?

- For men, the most common site of melanoma is the back.
- For women, the most common site of melanoma is the legs.
- Melanoma can occur in the eye (ocular melanoma).

Moles normally resemble each other, so it helps to look out for any moles that look different to the surrounding ones. This is known as the **‘ugly duckling sign’**. If a melanoma develops, it may look and feel different and may also change differently to the moles near it. Other changes to look out for include:

- **Change in shape or colour**
- **Increase in size**
- **Bleeding or itching**
- **Irregular border**
- **New moles or freckles**

Look out for the ‘ugly duckling sign’

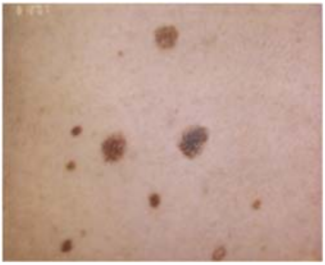

Where to look:

Head, scalp, neck & ears: Use your hand or the mirror, or get someone to look for you.

Torso: Front, back, then right and left sides with your arms raised.

Arms, hands, fingers & nails: Look carefully at forearms and upper underarms.

Buttocks & legs: Use a mirror or get someone to help you.

Feet, including soles and toes

Examples of melanoma:

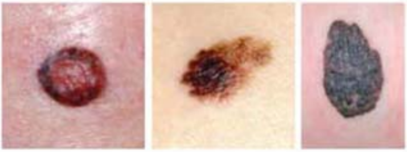

The Cancer Council’s **SunSmart** website gives tips for checking your skin and includes an online video showing a step-by-step approach to skin self-examination:

www.sunsmart.com.au/skin-cancer/checking-for-skin-cancer

3 Have a general practitioner or dermatologist check your skin for melanoma

Doctors use a number of tools and techniques to examine skin thoroughly, beyond what the naked eye can see.

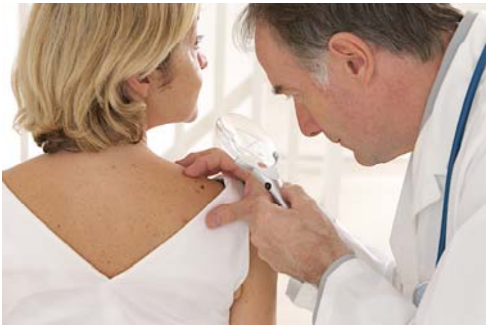

Melanomas that are detected and treated early are cured in 90% of cases.

It is important to get a professional skin check by a doctor if anything suspicious appears on your skin.

Should my children and other family members also have skin checks?

Melanoma is rare before puberty. For this reason, younger children usually only have their skin examined by a doctor if their parent is concerned about a particular spot or mole.

While teenagers can develop melanoma, their risk is much lower than adults. Regular skin checks by a doctor in adolescence would usually only occur when other risk factors are present, such as a strong family history of melanoma.

All adults should be familiar with checking their own skin, and should talk to their GP about how often to get a skin check by a health professional .

Where can I get my skin checked?

A GP can perform a skin check and examine any lesions of concern. They are familiar with your history, can talk to you about other risk factors, and can treat some skin cancers. They might also refer you to a dermatologist, if needed.

Skin Cancer Clinics

There are many skin cancer clinics across Australia. Skin cancer clinics are usually operated by GPs, but their focus is on skin cancer.

Dermatologists

For a second opinion, or for people at high risk of melanoma, a referral to see a dermatologist is recommended.

Dermatologists are doctors who have completed additional training to specialise in diagnosing and treating skin disease, including skin cancers.

To see a dermatologist, you will need a referral from a GP or a skin cancer clinic. Keep in mind that there can be long waiting times to get an appointment, and also be sure to ask about the fees and what is covered by Medicare.

How much sun do I need to maintain adequate Vitamin D levels?

Ultraviolet (UV) radiation from the sun is one of the main causes of melanoma, but it is also one of the best sources of Vitamin D. In Australia we need to balance the need for sun protection with our body's need for Vitamin D. Some sun exposure is still important because it allows your body to make Vitamin D, which is essential for good bone health and may have other health benefits. Small amounts of Vitamin D can also be found in foods such as oily fish (salmon, herring and mackerel), liver, eggs and some dairy products. Vitamin D supplements are also available.

In most areas of Australia, for people with fair skin, you only need 6 to 8 minutes of direct sunlight a day before 10am or after 2pm, to get enough vitamin D. In southern parts of Australia during winter you need 30 to 50 minutes of direct sunlight a day before 10am or after 2pm.

Most Australians get all the sun they need from everyday outdoor activities such as walking to the local shops, waiting for a bus, hanging out the washing, or walking their children to school. Using sensible sun protection when outdoors does not put you at risk of developing vitamin D deficiency. Talk to your doctor if you are concerned about your vitamin D levels.

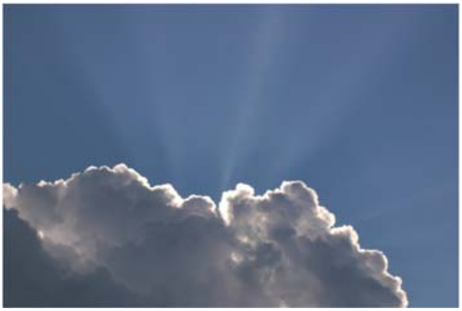

Other resources

Further information about melanoma can be accessed via the organisations listed below:

Cancer Council Australia Helpline: 13 11 20

The Cancer Council Helpline is a confidential telephone service for emotional support and detailed information about any type and aspect of cancer. Ringing the Helpline costs the same as a local call. The Helpline is staffed by experienced cancer health professionals including oncology nurses, counsellors and a radiation therapist. You can access the Helpline Monday to Friday, from 9am to 5pm AEST. Outside these hours, you can leave a message and your call will be returned. The Cancer Council NSW Helpline is available in 120 languages and dialects via the **National Translation and Interpreting Service (TIS).** For the cost of a local call, contact the TIS on 13 14 50. You can visit the Cancer Council NSW website at: **www.cancercouncil.com.au**

Melanoma Patients Australia Support Help Line: 1300 88 44 50

Melanoma Patients Australia is a not-for profit, patient-driven organisation that provides support and information about melanoma prevention, diagnosis, management and treatment for melanoma patients, their families, carers, and friends. The website gives you a lot of information on melanoma, and also provides a lot of information on support, awareness and advocacy for melanoma patients and their families. **http:/www.melanomapatients.org.au/**

mpa
Melanoma Patients Australia

Cancer Council NSW

Melanoma Institute Australia

Melanoma Institute Australia is a not-for-profit organisation involved in preventing and curing melanoma through research, treatment and education programs. There is a lot of information about melanoma, including the causes, prevention, early detection and screening on this website that you may find useful. **http://www.melanoma.org.au/**

SunSmart

The SunSmart program is an initiative of Cancer Council Victoria. SunSmart programs run in all Australian states and territories by the Cancer Council. SunSmart provides multiple resources for skin cancer prevention and sun protection that you may find useful **http://www.sunsmart.com.au**

ARPANSA

ARPANSA has a network of UV detectors in cities around Australia. The website provides you with more detailed information about UV levels in Australia **http://www.arpansa.gov.au/uvindex/realtime/index.cfm**

Disclaimer: While the information in this booklet has been prepared with all due care, changes after the time of printing may impact the accuracy of information. Links to Internet sites and other organisations are provided for information only. Care has been taken in providing these links as suitable reference resources. However, due to the changing nature of the Internet, it is the responsibility of users to make their own decisions and enquiries about the information retrieved from Internet sites or other organisations. Date of printing: 13 October 2017

Acknowledgements: The study research team would like to acknowledge information obtained from other sources including SunSmart, Cancer Councils, GenoMEL (the international melanoma genetics consortium), Dr Nadine Kasparian, the PennSCAPE Study (Skin Cancer Awareness, Prevention and Education), Melanoma Institute of Australia, and the American Academy of Dermatology. Our study received funding from the National Health and Medical Research Council (NHMRC). Some members of the research team receive funding for salary support from the NHMRC and the Cancer Institute NSW.
